# Efficacy and safety of eight types *Salvia miltiorrhiza* injections in the treatment of unstable angina pectoris: A network meta-analysis

**DOI:** 10.3389/fphar.2022.972738

**Published:** 2022-10-03

**Authors:** Mingxuan Li, Hongdian Li, Hongxu Liu, Xiaolei Lai, Wenlong Xing

**Affiliations:** ^1^ Beijing Hospital of Traditional Chinese Medicine, Capital Medical University, Beijing, China; ^2^ Beijing University of Chinese Medicine, Beijing, China

**Keywords:** Traditional Chinese Medicine, unstable angina (UA), network meta-analysis, Salvia miltiorrhiza injection classes, Chinese medicine injection

## Abstract

**Background:** *Salvia miltiorrhiza Bunge*. [Lamiaceae, danshen] injection classes (SMIC) is widely used in the treatment of unstable angina (UA). However, it is uncertain which SMIC is more effective in terms of UA efficacy. The purpose of this Network Meta-analysis (NMA) was to compare the treatment effects of various SMIC to determine the best SMIC for the treatment of UA.

**Methods:** The China National Knowledge Infrastructure (CNKI), Wanfang Database, China Science and Technology Journal Database (VIP), Chinese Biomedical Literature Database (CBM), PubMed, Web of Science, and Cochrane Library databases were searched to screen randomized controlled trials (RCTs) of SMIC for UA. The search time frame was all from the establishment of the database to May 2022. RevMan 5.3 and Stata 14.0 software were used for NMA.

**Results:** A total of 148 studies including 14,979 patients, including 7,584 cases in the experimental group and 7,395 cases in the control group were included, and eight SMIC were extracted, namely:Danshen injection, Fufang Danshen injection, Guanxinning injection, Danshenchuanxiongqin injection, Danhong injection, Danshentong IIA Huangsuanna injection, Shenxiong Putaotang injection, and Danshenduofensuanyan injection. The results of NMA showed that, in terms of total effective rate, Shenxiong Putaotang injection and Danshenchuanxiongqin injection have the advantage; In terms of ECG efficiency, Danshentong IIA Huangsuanna injection and Danshen injection have an advantage; Danshenchuanxiongqin injection and Danshenduofensuanyan injection were more effective than other SMIC in improving angina pectoris attacks; Shenxiong Putaotang injection has an advantage in improving hs-CRP; Shenxiong Putaotang injection and Danshentong IIA Huangsuanna injection have advantages in improving TC and TG, respectively.

**Conclusion:** The eight SMIC included in the current study were effective in treating UA, Shenxiong Putaotang injection and Danshentong IIA Huangsuanna injection were both superior in improving all outcome indicators. However, there is still a need for larger samples and high-quality randomized controlled trials for more refined comparisons of various SMIC.

**Systematic Review Registration**: [PROSPERO], identifier [CRD42022350872]

## 1 Introduction

The China Cardiovascular Health and Disease Report 2020 (National Center for Cardiovascular Disease, 2021) shows that the prevalence of cardiovascular disease in China is on the rise, and it is estimated that the number of people suffering from cardiovascular disease is about 330 million, including about 11.39 million with coronary heart disease. Unstable angina (UA) is a clinical state between stable angina (SA) and acute myocardial infarction (AMI), and is a common type of acute coronary syndrome (ACS) (Prisant et al., 2017). Acute coronary syndrome encompasses unstable angina pectoris together with non-ST-elevation myocardial infarc-tion (NSTEMI) and ST-elevation myocardial infarction (STEMI) (Braunwald et al., 2000; Braunwald and Morrow, 2013). Unstable angina has a higher annual morbidity and mortality rate compared to stable angina (Chen, 2007a). Plaque rupture is the main cause of unstable angina, mostly caused by increased plaque lesion activity and plaque fatigue; the risk of thrombosis is greatly increased when the plaque is exposed to high shear flow sites, and the thrombogenic effect of high cholesteryl ester ulcerated surfaces is most significant (Wei et al., 2017; Katus and Giannitsis, 2018; Sun et al., 2021). When active thrombosis is present in patients with unstable angina, it is mostly manifested by an increase in serum fibrin-associated antigen, D-dimer and fibrinopeptide concentrations, while in patients with stable angina, there are no such changes, which can lead to complete occlusion of the coronary artery with the gradual aggravation of this state, thus causing myocardial infarction and even sudden death (Pintova et al., 2021). ACC/AHA guidelines recommend routine use of UA medications including:Nitrates, β-Adrenergic Blockers, Calcium-Channel Blockers, Angiotensin-converting enzyme inhibitors (ACEIs)/angiotensin receptor blockers (ARBs), Antiplatelet agents, Glycoprotein IIb/IIIa Receptor Inhibitors (GPI), Anticoagulant therapy (Grosser et al., 2013; Amsterdam et al., 2014; Schjerning Olsen et al., 2015; Silvain et al., 2016). Therapeutic aims areto improve quality of life by decreasing anginal attacks and to prevent myocardial infarction and death. But not all of these drugs are useful in every form of UA, and treatmentis symptomatic rather than curative (Zavecz and Bueno, 1995; Yang et al., 2014a). The other drawbacksof current anginal medications are adverse effects and drugresistance (Taggart, 2011). Treatments that effectively improve the prognosis of UA remain a challenging clinical challenge.

Chinese herbal medicine (CHM) has a 3,000-year-old history with unique theories for concepts of etiology and systems of diagnosis and treatment (Chen and Xu, 2003). It still has a very strong influence on medical practice in China and other countries around the world. There has been considerable interest in the benefits of CHM and potential drug interactions with western drugs, particularly in patients with UA (Eisenberg, 2011). Traditional Chinese Medicine (TCM) has a unique understanding of unstable angina pectoris, which belongs to the category of “stroke heartache” in TCM (Zhang et al., 2022). Many CHMs have been used to treat this disease from ancient times to the present, among which *Salvia miltiorrhiza Bunge*. [Lamiaceae, danshen] is one of the most widely used drugs. *Salvia miltiorrhiza* is derived from the dried root and rhizome of *Salvia miltiorrhiza* Bge. and has a long history of use in China as a traditional Chinese medicine (Song, 2018). *Salvia miltiorrhiza* contains a variety of chemical components, which are mainly divided into two categories: one is diterpene fat-soluble components, mainly tanshinone type diterpenes, and the other is water-soluble components, mainly phenolic acids. In addition, there are nitrogenous compounds, lactones, polysaccharides, flavonoids, steroids, triterpenes and other components (Wan et al., 2020). According to the theory of TCM, *Salvia miltiorrhiza* has the effect of stopping bleeding and pain, activating blood circulation and removing blood stasis, and anti-inflammatory (Wang, 2017a). It can protect cardiovascular, anti-arrhythmia, anti-atherosclerosis, improve microcirculation, protect myocardium, inhibit and release platelet aggregation, increase coronary flow, improve the body’s ability to resist hypoxia, anti-inflammatory, antioxidant, protect liver cells, anti-tumor and other biological activities (Zhao et al., 2007; Xu et al., 2021). In view of this, Chinese medicine injections (CMIs) with *Salvia miltiorrhiza* as the main ingredient are widely used to treat UA (Han and Wu, 2010; Gao et al., 2018; Wang et al., 2019a). Among the more commonly used SMIC are: Danshen injection (DS), compound Danshen injection (FFDS), coronary heart nin injection (GXN), Danshenchuanxiongqin injection (DSCXQ), and Danhong injection (DH), which are collectively known as *Salvia miltiorrhiza* injection classes (SMIC).

The results of a previous meta-analysis (Cui et al., 2012; Yang et al., 2012; Wu, 2013; Yao et al., 2013; Liu, 2015a; Niu et al., 2015; Yan et al., 2015; Chen et al., 2022) demonstrated that DS, DH, and GXN were used as adjuncts to conventional treatment of UA and were beneficial compared to conventional treatment alone in terms of for the total effective rate of UA, angina attacks, and improvement of inflammatory markers. However, it is not known which SMIC have better clinical outcomes. This has led to confusion among many medical practitioners regarding the choice of SMIC, potentially inadvertently causing patients to miss out on the best treatment options.

Therefore, we decided to use Network Meta-analysis, which combines direct comparative evidence in randomized controlled trials (RCTs) and indirect comparative evidence based on co-controlled RCTs (Cipriani et al., 2013), to try to find the most reliable certain SMIC for the treatment of UA through a network meta-analysis of relevant RCTs, as well as to assess the relative efficacy and safety of different SMIC in patients with UA. At present, there are more than 100 kinds of CMIs with national standards, among which there are more applications of cardiovascular Chinese medicine injections and about 30 varieties for the treatment of angina pectoris of coronary heart disease (Liu and Xie, 2020).

The present study was proposed to compare eight SMIC widely used in medical units at all levels in China, includin: Danshen injection (DS), Fufang Danshen injection (FFDS), Guanxinning injection (GXN), Danshenchuanxiongqin injection (DSCXQ), Danhong injection (DH), Danshentong IIA Huangsuanna injection (DST IIA HSN), Shenxiong Putaotang injection (SXPTT), Danshenduofensuanyan injection (DSDFSY). Combined treatment with CMI can not only quickly relieve the immediate symptoms such as palpitations, but also effectively relieve other accompanying symptoms such as insomnia, excessive dreaming and fatigue (Chen et al., 2015a).

## 2 Methods

### 2.1 Study registering and reportin

This programme is based on the Preferred Reporting Items for Systematic Reviews and Meta-Analyses Programme (PRISMA-P) (Shamseer et al., 2015). The PRISMA Extension Statement is used to ensure that all aspects of the method and results are reported (Hutton et al., 2015).

### 2.2 Eligibility criteria

The PICOS (Population-Intervention-Comparators-Outcomes-Study design) framework was adopted as the eligibility criteria for the review as following.

#### 2.2.1 Study design

RCTs on SMIC for the treatment of unstable angina, blinded and in any language.

#### 2.2.2 Population

All patients enrolled were receiving regular treatment for unstable angina (e.g., Nitrates, β-Adrenergic Blockers, Calcium-Channel Blockers, Angiotensin-converting enzyme inhibitors (ACEIs)/angiotensin receptor blockers (ARBs), Antiplatelet agents, Glycoprotein IIb/IIIa Receptor Inhibitors (GPI), Anticoagulant therapy etc.), No other serious underlying disease, such as acute myocardial infarction, severe heart failure, coagulation disorders or liver or kidney disease, or malignancy, with an expected survival of less than 1 year; Western or Chinese medical diagnostic criteria must meet the accepted diagnostic criteria for unstable angina pectoris in coronary artery disease at the time of study publication; patients were enrolled regardless of age, gender, race, geography, disease duration, or ethnicity.

#### 2.2.3 Interventions/comparators

We decided to study the eight most commonly used SMIC through previous studies and actual clinical observations. These eight SMIC are: Danshen injection (DS), Fufang Danshen injection (FFDS), Guanxinning injection (GXN), Danshenchuanxiongqin injection (DSCXQ), Danhong injection (DH), Danshentong IIA Huangsuanna injection (DST IIA HSN), Shenxiong Putaotang injection (SXPTT), Danshenduofensuanyan injection (DSDFSY). For the purpose of data analysis, we defined conventional treatment as the administration of standardized treatment and symptomatic treatment consistent with the guidelines approved at the time the study was conducted. Eligible comparisons are as follows: control group, patients receiving regular treatment for unstable angina, without CMIs or Oral Chinese Medicine (OCM), etc., with unlimited duration of treatment and dose administered; appropriate treatment and symptomatic treatment for the patient’s own underlying disease condition is permitted. In the treatment group, only one of the above SMIC must be used in combination with the corresponding control group, and the dosage and duration of treatment must be in accordance with the specifications for the use of the drugs.

Studies that did not meet all of these inclusion criteria were excluded. In addition, the following exclusion criteria were applied:

①Repeated publications; ②Personal experience summary, pure theoretical research, and animal experiment; ③In addition to SMIC, the two groups also used traditional Chinese medicine decoction, Chinese patent medicine or acupuncture and moxibustion; ④ The number of reported cases in each group was too small (<20 cases); ⑤Too short treatment course (<7 days); ⑥The data in the literature are incorrect or incomplete.

### 2.3 Outcome measures

By reviewing clinical trials published in academic journals that evaluated UA, we decided to use evaluation parameters including: Total effective rate, According to the guidelines for UA and ECG efficacy criteria set by the Chinese Medical Association, the efficacy criteria are “significantly effective”, “effective” and “ineffective” (Liu et al., 1998; Department of Internal Medicine Society of Chinese Medicine Society, 2008). Electrocardiography is a valid and easy test regarding prognosis (Savonitto et al., 1999), and the evaluation criteria regarding the efficacy of electrocardiography are. Significantly effective: disappearance of angina symptoms and return to normal ECG examination. Effective: 50% reduction in the number of angina attacks per week compared to the pre-treatment period and a decrease in the ST segment to 0.02 mV or shallowing of the inverted T wave on ECG. Ineffective: no significant improvement in the condition (Ke and Chen, 2007). The frequency of angina attacks is a reliable indicator to evaluate the patient’s condition status, so the number of angina attacks per week or per day of the patient was included as one of the main observations. High-sensitivity CRP (hs-CRP) is significantly correlated with the occurrence of future adverse cardiovascular events and is a predictor of future cardiovascular events, which is significant for the development of cardiovascular disease (La Franca et al., 2013; Milwidsky et al., 2017); Lipid levels are closely related to the occurrence of intimal lipid deposition, which is one of the causes of the progression of atherosclerosis and is inseparable from the development of coronary artery disease (De Lau et al., 2008). It can be used to evaluate the effectiveness of SMIC in the treatment of UA patients.

In summary, Outcome indicators primary indicators: ① Total effective rate (Significantly effective + Effective), ② ECG efficacy (Significantly effective + Effective), ③ Angina pectoris attacks; secondary indicators: ① hs-CRP (High sensitivity C-reactive protein), ② total cholesterol (TC), ③ triglyceride (TG).

### 2.4 Data sources and search strategy

Computer search of seven databases: The China National Knowledge Infrastructure (CNKI), Wanfang Database, China Science and Technology Journal Database (VIP), Chinese Biomedical Literature Database (CBM), PubMed, Web of Science, and Cochrane Library databases. The search time of each database is built until May 2022. In addition, references to the literature were incorporated retrospectively to supplement access to relevant literature. The search was conducted with a combination of subject terms and free terms. English search terms included: “Coronary Diseases”, “Coronary Heart Disease”, “Unstable angina”, “Traditional Chinese medicine”, “Injection”, “Injectable”, “Danshen Injection”, “Randomized Controlled Trial” etc. Take PubMed as an example, the search terms and strategies are as follows. The search strategies of other databases were adjusted according to their search rules.

#1 Coronary Diseases [MeSH Terms]
#2 Coronary Diseases [Title/Abstract]
#3 Coronary Heart Disease [MeSH Terms]
#4 Coronary Heart Disease [Title/Abstract]
#5 #1 OR #2 OR #3 OR #4
#6 Unstable angina [MeSH Terms]
#7 Unstable angina [Title/Abstract]
#8 #6 OR #7
#9 Traditional Chinese medicine [MeSH Terms]
#10 Traditional Chinese medicine [Title/Abstract]
#11 Injection [MeSH Terms]
#12 Injection [Title/Abstract]
#13 Injectable [MeSH Terms]
#14 Injectable [Title/Abstract]
#15 Danshen Injection [MeSH Terms]
#16 Danshen Injection [Title/Abstract]
#17 #9 OR #10 OR #11 OR #12 OR #13 OR #14 OR #15 OR #16
#18 Randomized Controlled Trial [Publication Type]
#19 Controlled Clinical Trial [Publication Type]
#20 random [All Fields]
#21 #18 OR #19 OR #20
#22 #5AND #8 AND #17 AND #21

Two investigators (LMX and LHD) performed the literature screening and data extraction. After excluding duplicates, the titles and abstracts were read to exclude literature that clearly did not meet the requirements, and then the remaining literature was read in its entirety to clarify whether it met the inclusion criteria, and if there was disagreement, it was determined by discussion or consultation with a third investigators (XWL). Use Microsoft Excel 2019 to perform data extraction and collect relevant information. Data extraction included: ① Basic information: article title, first author, publication date, country/region, etc.; ② Tudy characteristics: interventions in the trial and control groups, number of study subjects, age, duration of intervention, adverse effects; ③ Key information required for risk of bias evaluation in the literature; ④ Outcome indicators included in the trial and control groups.

### 2.5 Quality assessment

The study quality of the RCTs was assessed by two investigators using the tool for assessing risk of bias recommended in the Cochrane systematic reviewers’ handbook 5.1 (Higgins and Green, 2013). The following seven areas are included: random-sequence generation (selection bias); allocation concealment (selection bias); blinding of participants and personnel (performance bias); blinding of outcome assessment (detection bias); incomplete outcome data (attrition bias); selective reporting (reporting bias); others bias. Each aspect can be further categorized as “low risk of bias” “unclear risk of bias” “high risk of bias” which can be decided by mutual discussion or consultation with a third investigator (XWL) when there is a difference of opinion.

### 2.6 Statistical analysis

Stata 14.0 (MRC Biostatistics Unit, Cambridge, United Kingdom) software was used to implement the statistical process. The mean difference (MD) was used as the effect analysis statistic for the measures, and the risk ratio (RR) was used as the effect analysis statistic for the dichotomous variables, with each effect providing its 95% confidence interval (CI). NMA was performed using the network group command in Stata 14.0 for data preprocessing, and evidence network plots were drawn to demonstrate the network relationships of different interventions, with the larger area of the dot in the plot indicating the greater number of patients for that intervention, and the thickness of the line connecting the two interventions representing the number of included studies (Zhang TS, 2015; Tian and Li, 2017). Two-by-two comparisons of different interventions were conducted, and inconsistency tests were performed when there was a closed loop, and the inconsistency factors (IF), 95% *CI* and the *p* value of its *Z* test were calculated, and when *p* > 0.05 and the lower limit of the 95% *CI* of IF value was equal to 0 were considered as good consistency between the results of direct and indirect comparisons, otherwise the closed loop was considered to have significant inconsistency, but according to this case there is no closed loop, so the consistency model was used. The efficacy was ranked according to surface under the cumulativeranking curve (SUCRA) (Yi et al., 2015), and the results of the final reticulated NMA were presented in tabular form to obtain the relatively best interventions. The “calibration-comparison” funnel plot was drawn to identify the presence of small sample effects and to assess publication bias.

Considering the inclusion of multiple observations, we combined the individual observations two by two and used a multivariate approach to determine the dependencies between the results. Clustering methods and two-dimensional graphs are used to generate the processing clusters (Chaimani et al., 2013). Using the “Clustering” command, a clustering ranking graph can be obtained using the Stata program. The different colors correspond to the estimated clusters and are used to group the treatments according to the similarity of the two results.

## 3 Results

### 3.1 Literature screening process and basic characteristics

**FIGURE 1:**
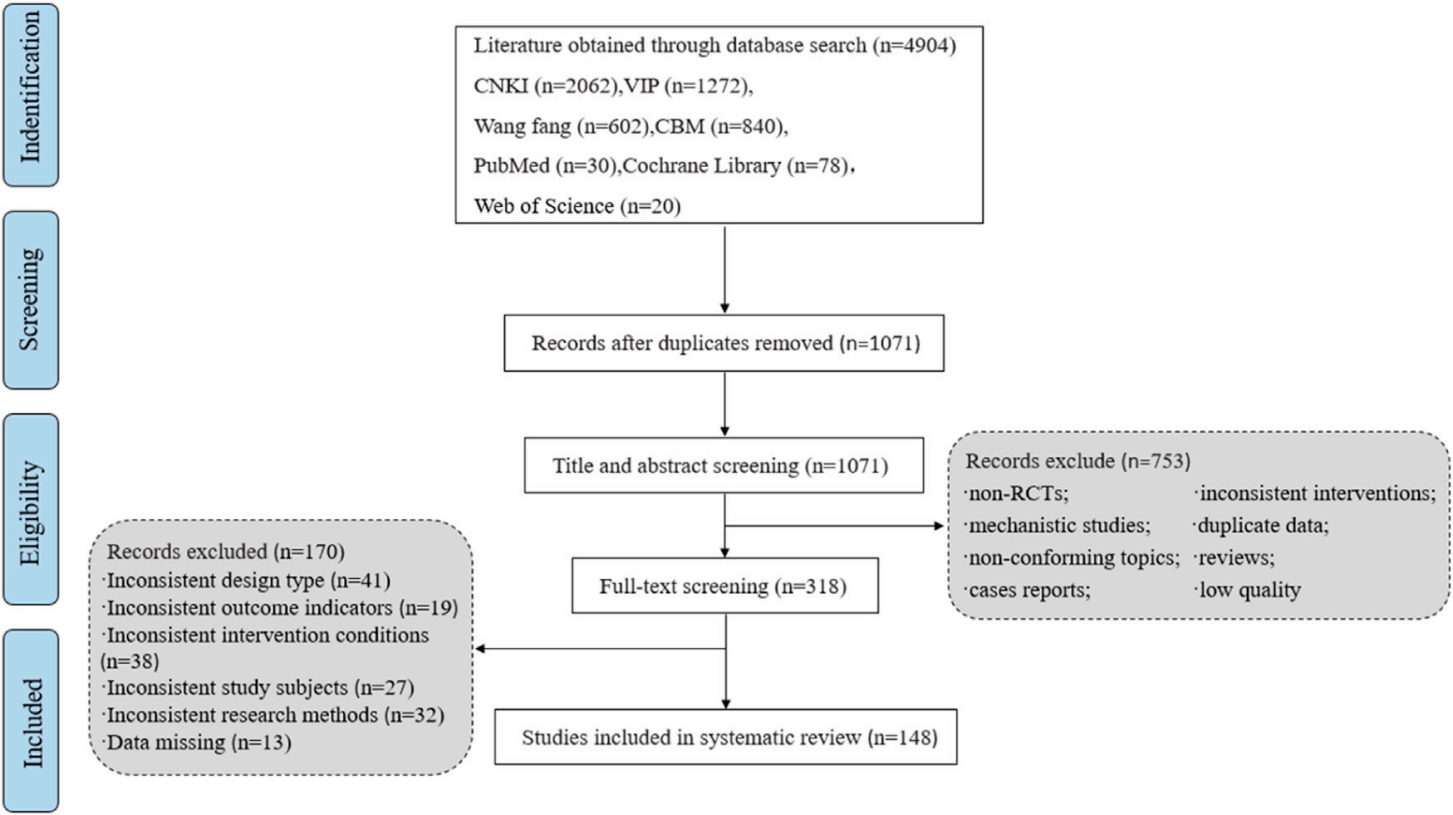
Flowchart of study selection.

A total of 4,904 literature were obtained by searching relevant databases. After excluding duplicate literature, 1,071 articles were obtained. By reading the titles and abstracts of the literature, 753 articles that obviously did not meet the inclusion criteria were excluded; for the remaining 318 articles, by reading the full text, 170 articles that did not meet the inclusion criteria in terms of study subjects, design types, and intervention conditions were excluded, and 148 RCTs studies were finally included (Ren, 2017; Li, 2006; Wang and Wu, 2013; Qian et al., 2000; Chen, 2007b; Zhao, 2013; Wang and Guo, 2009; Huang, 2014a; Zhou, 2010; Wu, 2019; Li, 2013; Yang and Ma, 2008; Tian and Wu, 2006; Hao and Wang, 2013; Li and Zhu, 2012; Ma and Peng, 2018; Tang, 2015; Song, 2014; Meng et al., 2014; Wang et al., 2011; Reng et al., 2012; Yu, 2017; Zhang and Lu, 2010; Xu and Hao, 2022; Cai et al., 2014; Liu et al., 2015a; Li et al., 2018; Zhang et al., 2019; Jiang, 2019; Ren et al., 2018; Jiang et al., 2014a; Yang et al., 2014b; Li, 2014a; Gu, 2019; Lan, 2015; Cai et al., 2013; Li, 2018a; Huang, 2017; Hu and Jia, 2014; Zhang, 2017; Yao, 2015; Li and Zhang, 2018; Yu, 2018; Zhao et al., 2015; Huang, 2015; Ma, 2015a; Peng et al., 2015; Yang et al., 2016; Pu, 2017; Wang, 2018; Pi et al., 2019; Lv, 2016; Pu, 2014; Dai, 2015; Chang, 2021; Yang, 2019; Duan, 2019; Liu and Zhang, 2011; Xia, 2011; Pu et al., 2010; Liu, 2015b; Yu and Xu, 2021; Jin and Han, 2020; Zhang HL, 2015; Liang and Dong, 2014; Huang, 2014b; Wang, 2012; An, 2010; Pan, 2012; Lu and Liang, 2015; Yang, 2017a; Liu, 2016; Kang and Hou, 2015; Li, 2018b; Li, 2014b; Li and Lin, 2014; Wang and He, 2011; Xu and Hu, 2016; Zhang, 2016; Jiang et al., 2014b; Huang et al., 2013; Zhao et al., 2012; Du, 2018; Li et al., 2016; Han and Wang, 2011; Chen, 2014; Fang et al., 2016; Guo, 2021; Hu, 2010; Xi et al., 2015; Li and Zhou, 2015; He, 2012; Qin et al., 2010; Chen, 2012; Liang et al., 2011; Luo and Kang, 2011; Qi and Wang, 2011; Liu and Xiao, 2014; Wu et al., 2016; Mao et al., 2016; Chen et al., 2016; Chen et al., 2014; Ren et al., 2012; Han et al., 2016; Liu, 2017; Xia et al., 2016; Zhao et al., 2018; Xu et al., 2013; Li et al., 2014a; Jiao and Wang, 2019; Yldx and As, 2016; Dou, 2010; Liu, 2015c; Yin and Xu, 2013; Qin, 2014; Xu et al., 2011; Kang et al., 2012). The literature screening process is shown in Figure 1, 148 studies were all in Chinese, with a total of 14,979 patients, including 7,584 in the experimental group and 7,395 in the control group. EightSMIC were included, specifically: Danshen injection (6 items), Fufang Danshen injection (5 items), Guanxinning injection (7 items), Danshenchuanxiongqin injection (23 items), Danhong injection (55 items), Danshentong IIA Huangsuanna injection (9 items), Shenxiong Putaotang injection (10 items), Danshenduofensuanyan injection (33 items). The basic characteristics of the included studies are shown in Table 1.

**TABLE 1:** Characteristics of included studies.

Included studies	Sample (E/C)	Sex (M/F)		Age		Disease duration		Intervention		Time	Outcomes
		E	C	E	C	E	C	E	C		
Ren, (2017)	48/47	29/19	27/20	60.8 ± 6.7	61.6 ± 6.9	5.5 ± 2.3	5.6 ± 2.1	Danshen injection + CT	CT	4w	①⑦
Li, (2006)	85/83	53/32	49/34	59.9 ± 3.25	58.6 ± 3.14	—	—	Danshen injection + CT	CT	10 d	①②
Wang and Wu, (2013)	42/42	23/19	22/20	46–67	46–68	—	—	Danshen injection + CT	CT	10 d	①
Qian et al. (2000)	32/27	25/7	22/5	69.2	70	2.2 m	2.1 m	Danshen injection + CT	CT	14 d	①
Chen, (2007b)	45/35	24/21	18/17	65 ± 6.21	66 ± 7.28	>8 w	>6 w	Danshen injection + CT	CT	14 d	①⑦
Zhao, (2013)	34/32	20/14	18/14	63	65	—	—	Danshen injection + CT	CT	14 d	①
Wang and Guo, (2009)	40/40	28/12	27/13	66	65	—	—	Fufang Danshen injection + CT	CT	15 d	①②⑦
Huang, (2014a)	40/40	20/20	21/19	64.5 ± 6.3	63.1 ± 7.3	8.2 ± 1.5	8.3 ± 1.4	Fufang Danshen injection + CT	CT	28 d	①③
Zhou, (2010)	72/65	35/37	31/34	60.5 ± 18.6	61.5 ± 20.3	—	—	Fufang Danshen injection + CT	CT	15 d	①②⑦
Wu, (2019)	77/77	45/32	40/37	47.5 ± 2.4	48.7 ± 1.2	—	—	Fufang Danshen injection + CT	CT	14 d	①⑦
Li, (2013)	57/57	32/25	30/27	67.4 ± 3.9	65.1 ± 5.9	3.1 ± 0.6	3.3 ± 0.5	Fufang Danshen injection + CT	CT	10 d	①
Yang and Ma, (2008)	50/40	35/15	26/14	66.5	66.1	—	—	Guanxinning injection + CT	CT	14 d	①②
Tian and Wu, (2006)	32/30	15/17	16/14	62.4 ± 4.6	61.3 ± 5.8	11.6 ± 3.4	8.4 ± 2.9	Guanxinning injection + CT	CT	14 d	①③
Hao and Wang, (2013)	29/29	18/11	19/10	65.7 ± 5.6	65.3 ± 5.2	—	—	Guanxinning injection + CT	CT	15 d	①⑦
Li and Zhu, (2012)	34/34	20/14	23/11	61.46 ± 9.92	64.4 ± 7.81	7.45 ± 6.54	7.96 ± 3.96	Guanxinning injection + CT	CT	10–14 d	①②
Ma and Peng, (2018)	60/60	40/20	41/19	66.8	66.7	—	—	Guanxinning injection + CT	CT	2w	①⑦
Tang, (2015)	30/30	24/9	18/9	65.5	64.3	—	—	Guanxinning injection + CT	CT	2w	①②
Song, (2014)	48/38	32/16	25/13	66.5 ± 2.12	66.1 ± 2.09	—	—	Guanxinning injection + CT	CT	15 d	①②
Meng et al. (2014)	51/51	32/19	31/20	62.2 ± 10.5	61.7 ± 9.9	—	—	Danshenchuanxiongqin injection + CT	CT	7 d	①②
Wang et al. (2011)	70/70	40/30	38/32	65.4 ± 7.8	64.7 ± 8.2	4.3 ± 1.2	4.4 ± 2.0	Danshenchuanxiongqin injection + CT	CT	14 d	①③⑤⑥
Reng et al. (2012)	60/60	39/21	37/23	60.6 ± 14.8	58.8 ± 15.3	—	—	Danshenchuanxiongqin injection + CT	CT	14 d	①⑤⑥⑦
Yu, (2017)	43/43	25/18	27/16	61.85 ± 10.08	62.09 ± 13.16	4.21 ± 1.03	4.17 ± 0.64	Danshenchuanxiongqin injection + CT	CT	14 d	①③⑦
Zhang and Lu, (2010)	25/25	14/11	16/9	61.6 ± 15.8	60.8 ± 16.3	—	—	Danshenchuanxiongqin injection + CT	CT	10 d	①②③
Xu and Hao, (2022)	30/30	17/13	20/10	65.75 ± 4.76	65.73 ± 4.88	11.50 ± 4.15	10.38 ± 3.45	Danshenchuanxiongqin injection + CT	CT	4w	③
Cai et al. (2014)	54/52	30/24	29/23	77 ± 6.8	76 ± 7.2	—	—	Danshenchuanxiongqin injection + CT	CT	15 d	①⑤⑥④⑦
Liu et al. (2015a)	62/60	34/28	32/28	67.7 ± 4.3	68.8 ± 5.1	—	—	Danshenchuanxiongqin injection + CT	CT	4w	①②⑤⑥④⑦
Li et al. (2018)	40/40	23/17	24/16	66.2 ± 6.1	65.9 ± 6.0	6.4 ± 2.9	6.0 ± 3.0	Danshenchuanxiongqin injection + CT	CT	1 m	①②④
Zhang et al. (2019)	59/58	42/17	43/15	59.1 ± 7.3	59.8 ± 7.7	5.7 ± 3.7	5.3 ± 3.4	Danshenchuanxiongqin injection + CT	CT	4w	①②③⑤⑥
Jiang, (2019)	23/23	9/14	8/15	55.32 ± 3.14	56.33 ± 5.49	4.23 ± 1.33	4.13 ± 1.14	Danshenchuanxiongqin injection + CT	CT	3 m	①④
Ren et al. (2018)	54/54	32/22	29/25	54.97 ± 11.29	53.92 ± 12.78	7.9 ± 6.2	7.1 ± 5.9	Danshenchuanxiongqin injection + CT	CT	14 d	①②③
Jiang et al. (2014a)	78/82	50/28	52/30	63.5	64.6	—	—	Danshenchuanxiongqin injection + CT	CT	14 d	①②⑦
Yang et al. (2014b)	72/65	42/30	38/27	55 ± 18.9	51 ± 19.3	—	—	Danshenchuanxiongqin injection + CT	CT	15 d	①③⑦
Li, (2014a)	40/40	21/19	20/20	61.6 ± 15.8	60.8 ± 16.3	—	—	Danshenchuanxiongqin injection + CT	CT	10 d	①②
Gu, (2019)	68/68	41/27	39/29	57.41 ± 5.52	55.68 ± 4.58	4.85 ± 1.35	4.82 ± 2.53	Danshenchuanxiongqin injection + CT	CT	3w	①④
Lan, (2015)	116/100	67/49	57/43	57.6 ± 4.6	58.1 ± 5.2	7.62 ± 3.87	8.19 ± 4.28	Danshenchuanxiongqin injection + CT	CT	14 d	①②⑦
Cai et al. (2013)	63/58	38/25	35/23	76 ± 4.6	75 ± 5.2	0–14 y	0.08–12 y	Danshenchuanxiongqin injection + CT	CT	14 d	①②⑦
Li, (2018a)	200/200	100/100	104/96	56.43 ± 4.83	56.95 ± 4.15	7.35 ± 1.83	7.98 ± 2.02	Danshenchuanxiongqin injection + CT	CT	2w	①
Huang, (2017)	50/50	26/24	28/22	54.39 ± 3.41	56.43 ± 3.21	—	—	Danshenchuanxiongqin injection + CT	CT	2w	③⑦
Hu and Jia, (2014)	38/38	20/18	21/17	54.72 ± 4.38	55.14 ± 4.63	6.12 ± 1.54	6.58 ± 1.74	Danshenchuanxiongqin injection + CT	CT	2w	①③⑤⑥
Zhang, (2017)	40/40	19/21	22/18	55.4 ± 2.6	55.7 ± 2.4	7.3 ± 2.6	7.9 ± 2.2	Danshenchuanxiongqin injection + CT	CT	2w	①③
Yao, (2015)	70/70	33/37	34/36	56.46 ± 3.80	57.43 ± 3.51	5.37 ± 0.41	5.65 ± 0.48	Danshenchuanxiongqin injection + CT	CT	10 d	①⑤⑥
Li and Zhang, (2018)	60/60	36/24	35/25	75.8 ± 4.5	75.2 ± 4.9	4.4 ± 0.8	4.2 ± 0.5	Danhong injection + CT	CT	30 d	①③⑦
Yu, (2018)	28/28	17/11	14/14	62.35 ± 2.62	64.45 ± 3.07	—	—	Danhong injection + CT	CT	14 d	①
Zhang et al. (2014)	56/56	43/13	42/14	62.2	62.3	—	—	Danhong injection + CT	CT	2w	①②⑦
Xu and Liu, (2011)	45/40	20/25	19/21	64.5 ± 4.28	63.2 ± 5.15	—	—	Danhong injection + CT	CT	14 d	①③
Bian, (2015)	50/48	27/23	22/26	57.9 ± 6.6	55.8 ± 4.8	29.8 ± 10.5 m	27.5 ± 9.8 m	Danhong injection + CT	CT	14 d	①②⑦
Huang et al. (2015)	37/37	21/16	22/15	56.7 ± 1.3	56.9 ± 1.4	5.2 ± 0.7	5.1 ± 0.6	Danhong injection + CT	CT	10 d	①⑦
Liu et al. (2015b)	52/52	32/20	34/18	61.5 ± 9.1	63.8 ± 9.9	4.5 ± 1.8	4.2 ± 2.0	Danhong injection + CT	CT	2w	①④
Zhao et al. (2015)	54/54	27/27	26/28	61.93 ± 4.41	62.53 ± 4.73	4.23 ± 2.41	4.83 ± 2.51	Danhong injection + CT	CT	2w	①④
Huang, (2015)	51/51	26/25	28/23	63.3 ± 5.4	66.3 ± 5.8	—	—	Danhong injection + CT	CT	10 d	①
Ma, (2015a)	40/40	24/16	23/17	56.41 ± 4.52	55.64 ± 4.23	4.62 ± 1.34	4.82 ± 2.13	Danhong injection + CT	CT	14 d	①④
Peng et al. (2015)	45/45	26/19	27/18	63.2 ± 9.2	63.5 ± 9.0	—	—	Danhong injection + CT	CT	2w	①④
Yang et al. (2016)	34/31	21/13	19/12	63.47 ± 9.83	62.93 ± 9.49	—	—	Danhong injection + CT	CT	10 d	②⑦
Pu, (2017)	40/40	16/24	15/25	61 ± 4	61 ± 4	6.1 ± 0.7	5.8 ± 0.9	Danhong injection + CT	CT	2w	①②④⑤⑥
Wang, (2018)	41/41	17/24	15/26	58 ± 6	58 ± 6	7.2 ± 0.6	7.1 ± 0.7	Danhong injection + CT	CT	2w	①②⑤⑥
Pi et al. (2019)	54/54	29/25	28/26	67.03 ± 3.69	66.87 ± 3.76	4.23 ± 1.35	4.03 ± 1.25	Danhong injection + CT	CT	14 d	①②
Lv, (2016)	65/65	38/27	34/31	62.4 ± 3.7	64.4 ± 4.0	4.9 ± 1.2	3.4 ± 1.1	Danhong injection + CT	CT	15 d	①③④⑥⑦
Pu, (2014)	30/30	15/15	17/13	63.9 ± 12.4	65.2 ± 17.1	7.22 ± 1.21	7.19 ± 1.42	Danhong injection + CT	CT	14 d	①③
Dai, (2015)	40/40	22/18	21/19	65.4 ± 2.8	66.0 ± 3.0	-	-	Danhong injection + CT	CT	2w	①③
Chang, (2021)	43/43	23/20	23/20	66.21 ± 4.01	65.23 ± 3.45	10.12 ± 5.03	9.88 ± 3.65	Danhong injection + CT	CT	4w	①③
Yang, (2019)	50/50	28/22	27/23	63.10 ± 5.48	62.31 ± 6.01	-	-	Danhong injection + CT	CT	1 m	①
Duan, (2019)	63/63	34/29	35/28	63.25 ± 3.47	62.63 ± 3.58	5.17 ± 1.28	5.24 ± 1.35	Danhong injection + CT	CT	2w	①③
Liu and Zhang, (2011)	53/50	35/28	27/23	57 ± 5	56 ± 7	1 ± 0.5	1 ± 0.42	Danhong injection + CT	CT	14 d	①②③
Xia, (2011)	40/40	27/13	23/17	56.2 ± 7.8	57.3 ± 8.1	—	—	Danhong injection + CT	CT	10 d	①②
Pu et al. (2010)	45/42	25/20	24/18	75–91	74–89	—	—	Danhong injection + CT	CT	2w	②③
Liu, (2015b)	46/46	26/20	25/21	74.5 ± 3.7	74.3 ± 3.8	—	—	Danhong injection + CT	CT	2w	①③
Yu and Xu, (2021)	36/36	25/11	24/12	57.2 ± 13.2	56.8 ± 12.1	—	—	Danhong injection + CT	CT	2w	⑤⑥
Jin and Han, (2020)	40/40	25/15	26/14	66.01 ± 5.88	65.02 ± 5.78	3.09 ± 1.11	3.12 ± 1.13	Danhong injection + CT	CT	2w	①
Zhang HL (2015)	90/90	59/31	60/30	54 ± 3.5	57 ± 5.2	15.6 ± 2.4	16.4 ± 3.6	Danhong injection + CT	CT	7–14 d	①②③
Liang and Dong, (2014)	50/50	26/24	27/23	57.9 ± 10.6	56.9 ± 10.7	—	—	Danhong injection + CT	CT	2w	①
Huang, (2014b)	84/84	54/30	52/32	59.8 ± 3.68	62.2 ± 3.55	—	—	Danhong injection + CT	CT	2w	①⑦
Wang, (2012)	30/30	19/11	18/12	62.4	63.1	4–9	3–9	Danhong injection + CT	CT	15 d	①③
An, (2010)	41/41	28/13	29/12	61 ± 7.2	60 ± 6.0	—	—	Danhong injection + CT	CT	2w	①⑤⑥
Pan, (2012)	58/58	30/28	32/26	61	61.5	5.2	5.4	Danhong injection + CT	CT	14 d	①⑤⑥
Lu and Liang, (2015)	60/60	36/24	32/28	62.4	62.5	—	—	Danhong injection + CT	CT	2w	①②
Yang, (2017a)	53/53	38/15	39/14	41–76	42–77	2–8	3–7	Danhong injection + CT	CT	7–10 d	①②
Liu, (2016)	75/75	46/29	45/30	58.13 ± 7.68	59.05 ± 6.97	6.41 ± 2.48	6.47 ± 2.42	Danhong injection + CT	CT	2w	①③
Kang and Hou, (2015)	60/60	32/28	29/31	62.5 ± 5.4	62.8 ± 6.1	—	—	Danhong injection + CT	CT	14 d	①②④
Li, (2018b)	40/39	31/9	29/10	60.25 ± 8.31	60.33 ± 9.14	—	—	Danhong injection + CT	CT	1 m	①
Li, (2014b)	60/60	48/12	45/15	64.7	63.8	—	—	Danhong injection + CT	CT	3w	①③
Li and Lin, (2014)	40/40	28/12	30/10	62	63.5	—	—	Danhong injection + CT	CT	14 d	①②
Wang and He, (2011)	50/45	26/24	25/20	62 ± 4.7	60 ± 5.4	—	—	Danhong injection + CT	CT	2w	①②⑦
Xu and Hu, (2016)	60/60	38/22	31/29	59.5	60.5	6.12	6.09	Danhong injection + CT	CT	2w	①②
Zhang, (2016)	52/46	31/21	28/18	59.8 ± 9.3	54.4 ± 8.7	1.4 ± 0.2	1.3 ± 0.3	Danhong injection + CT	CT	14 d	①③⑦
Jiang et al. (2014b)	43/43	24/19	20/23	57.6 ± 13.5	59.1 ± 14.7	3.1 ± 2.0	2.9 ± 2.1	Danhong injection + CT	CT	2w	①②⑦
Xu and Meng, (2008)	29/26	19/10	17/9	68–75	69–78	—	—	Danhong injection + CT	CT	15 d	①②⑦
Xu and Lv, (2017)	66/62	30/36	32/30	69.4 ± 9.6	67.9 ± 7.6	2–12	3–14	Danhong injection + CT	CT	2w	①
Chen and Han, (2019)	55/55	26/29	28/27	70.6 ± 3.6	71.2 ± 3.5	3.4 ± 1.1	3.9 ± 1.2	Danhong injection + CT	CT	1w	①③⑤⑥
Hao et al. (2021)	49/49	28/21	26/23	69.84 ± 2.13	69.77 ± 2.09	3.39 ± 0.98	3.30 ± 0.89	Danhong injection + CT	CT	14 d	①⑦
Gong, (2014)	48/48	32/16	30/18	68.22 ± 6.44	69.78 ± 6.50	9.20 ± 4.38	9.32 ± 5.66	Danhong injection + CT	CT	14 d	①②
Wang, (2014)	32/32	23/9	22/10	48–77	48–75	3.8 ± 1.1	4.3 ± 1.3	Danhong injection + CT	CT	14 d	①②③⑦
Lai, (2012)	76/75	45/31	44/31	55.0 ± 1.50	56.00 ± 2.00	—	—	Danhong injection + CT	CT	14 d	①③
Ning et al. (2011)	25/25	16/9	15/10	62.4	63.1	4–9	3–9	Danhong injection + CT	CT	15 d	①③⑦
Gao et al. (2011)	80/80	39/41	43/37	59 ± 12.3	60 ± 10.9	—	—	Danhong injection + CT	CT	14 d	①②④
He and Sun, (2012)	48/48	32/16	30/18	60	62.5	—	—	Danhong injection + CT	CT	10 d	①②
Lv and Liu, (2011)	44/36	24/20	20/16	60.2 ± 2.4	62.4 ± 1.5	2–9	3–8	Danhong injection + CT	CT	14 d	①②③⑦
Ju, (2013)	30/30	18/12	16/14	64	62	—	—	Danshentong IIA Huangsuanna injection + CT	CT	10 d	①②⑦
Li and Xu, (2017)	48/48	26/22	28/20	68.2 ± 6.8	67.9 ± 6.5	—	—	Danshentong IIA Huangsuanna injection + CT	CT	10 d	①
Huang et al. (2013)	40/40	20/20	25/15	62.5	61.5	—	—	Danshentong IIA Huangsuanna injection + CT	CT	2w	①
Zhao et al. (2012)	40/40	20/20	21/19	68.75 ± 10.54	67.32 ± 10.23	4.66 ± 1.76	5.32 ± 1.37	Danshentong IIA Huangsuanna injection + CT	CT	14 d	①⑦
Du, (2018)	50/50	27/23	28/22	61.21 ± 2.62	61.32 ± 2.68	—	—	Danshentong IIA Huangsuanna injection + CT	CT	2w	①
Li et al. (2016)	51/51	30/21	29/22	62.5 ± 10.7	63.5 ± 10.7	—	—	Danshentong IIA Huangsuanna injection + CT	CT	2w	①
Han and Wang, (2011)	96/90	50/46	44/46	56	54	9	8	Danshentong IIA Huangsuanna injection + CT	CT	14 d	①
Chen, (2014)	86/86	56/30	54/32	55.5 ± 3.5	54.5 ± 3.0	11.5 ± 4.5	12.0 ± 4.0	Danshentong IIA Huangsuanna injection + CT	CT	2w	①⑤⑥⑦
Fang et al. (2016)	35/35	18/17	19/16	63.5 ± 10.3	62.2 ± 10.1	—	—	Danshentong IIA Huangsuanna injection + CT	CT	2w	①
Guo, (2021)	41/37	23/18	20/17	60.48 ± 9.81	60.23 ± 10.45	2.13 ± 0.65	2.07 ± 0.54	Shenxiong Putaotang injection + CT	CT	1 m	①③
Hu, (2010)	38/38	24/14	21/17	61	66	2–8	3–6	Shenxiong Putaotang injection + CT	CT	14 d	①④⑥
Xi et al. (2015)	35/35	21/14	17/18	55.3 ± 8.7	57.2 ± 10.3	2.1 ± 1.3	2.2 ± 1.2	Shenxiong Putaotang injection + CT	CT	3w	⑤⑥④
Li and Zhou (2015)	48/48	29/19	27/21	62.1 ± 5.8	63.2 ± 4.7	—	—	Shenxiong Putaotang injection + CT	CT	28 d	①③
He, (2012)	44/44	28/16	26/18	60.6 ± 6.8	62.2 ± 6.5	3 ± 1.83	3.17 ± 1.67	Shenxiong Putaotang injection + CT	CT	14 d	①⑤⑥④
Qin et al. (2010)	56/47	35/21	30/17	40–82	39–78	—	—	Shenxiong Putaotang injection + CT	CT	14 d	①②⑦
Chen (2012)	31/30	18/13	20/10	60.13 ± 7.86	59.03 ± 7.78	6.32 ± 4.55	7.10 ± 5.21	Shenxiong Putaotang injection + CT	CT	2w	①②⑦
Liang et al. (2011)	43/43	29/14	30/13	58.3	67.5	6.5	6.8	Shenxiong Putaotang injection + CT	CT	14 d	①⑦
Luo and Kang, (2011)	60/60	33/27	34/26	45–82	48–79	6.17	7.08	Shenxiong Putaotang injection + CT	CT	14 d	①②
Qi and Wang, (2011)	32/32	20/12	22/10	67.5 ± 9	66.8 ± 7.5	—	—	Shenxiong Putaotang injection + CT	CT	15 d	①②
Liu and Xiao, (2014)	83/77	48/35	45/32	65.3 ± 12.1	63.8 ± 11.3	—	—	Danshenduofensuanyan injection + CT	CT	4w	①②④
Wu et al. (2016)	48/48	27/21	26/22	61.35 ± 10.93	57.46 ± 10.20	—	—	Danshenduofensuanyan injection + CT	CT	8 d	①④
Mao et al. (2016)	48/48	32/16	31/17	58.3 ± 7.5	57.4 ± 7.3	—	—	Danshenduofensuanyan injection + CT	CT	2w	①④
Chen et al. (2016)	65/65	42/23	38/27	63.5 ± 7.2	62.7 ± 6.8	4.1 ± 2.2	4.2 ± 1.8	Danshenduofensuanyan injection + CT	CT	2w	①
Chen et al. (2014)	36/28	-	-	57.3 ± 5.6	56.9 ± 6.1	8.7 ± 1.9	9.1 ± 2.1	Danshenduofensuanyan injection + CT	CT	2w	①②⑦
Ren et al. (2012)	30/30	18/12	17/13	66.93 ± 9.58	61.85 ± 10.37	—	—	Danshenduofensuanyan injection + CT	CT	14 d	④⑦
Han et al. (2016)	49/49	14/11	15/10	70.9 ± 4.1	71.3 ± 4.8	3.3 ± 0.8	3.6 ± 0.4	Danshenduofensuanyan injection + CT	CT	2w	①⑤⑥
Liu, (2017)	40/40	23/17	22/18	59.12 ± 2.13	60.29 ± 2.24	—	—	Danshenduofensuanyan injection + CT	CT	2w	①⑦
Xia et al. (2016)	44/44	30/14	28/16	64.7 ± 10.1	63.1 ± 9.7	—	—	Danshenduofensuanyan injection + CT	CT	2w	①②③⑦
Zhao et al. (2018)	82/82	35/47	36/46	60.26 ± 1.80	59.71 ± 2.10	3.15 ± 0.91	3.87 ± 0.27	Danshenduofensuanyan injection + CT	CT	10 d	①⑦
Xu et al. (2013)	60/60	35/25	37/23	64.00 ± 6.98	63.37 ± 6.95	—	—	Danshenduofensuanyan injection + CT	CT	14 d	①②⑦
Li et al. (2014a)	92/92	50/42	50/42	58.3 ± 8.6	58.9 ± 8.9	—	—	Danshenduofensuanyan injection + CT	CT	2w	①②⑦
Jiao and Wang, (2019)	40/40	24/16	23/17	49.57 ± 4.39	49.63 ± 4.57	2.23 ± 1.09	2.27 ± 1.05	Danshenduofensuanyan injection + CT	CT	14 d	①⑦
Yldx and As, (2016)	40/40	22/18	25/15	58 ± 13	57.5 ± 10.5	—	—	Danshenduofensuanyan injection + CT	CT	14 d	①②
Dou, (2010)	46/46	26/20	25/21	65.2 ± 5	61.8 ± 4	—	—	Danshenduofensuanyan injection + CT	CT	14 d	①②
Liu, (2015c)	30/30	15/15	15/15	71.39 ± 2.48	71.37 ± 2.47	—	—	Danshenduofensuanyan injection + CT	CT	2w	①
Yin and Xu, (2013)	45/45	27/18	29/16	59.2 ± 8.6	58.8 ± 8.3	—	—	Danshenduofensuanyan injection + CT	CT	14 d	①②⑦
Qin, (2014)	30/30	15/15	16/14	59.23 ± 6.54	59.50 ± 6.48	6.1 ± 4.46	5.73 ± 4.39	Danshenduofensuanyan injection + CT	CT	14 d	①②⑦
Xu et al. (2011)	45/45	32/13	28/17	83.5 ± 3.5	81.2 ± 2.6	—	—	Danshenduofensuanyan injection + CT	CT	14 d	①②
Kang et al. (2012)	35/35	19/16	23/12	71.56 ± 8.25	73.77 ± 8.26	—	—	Danshenduofensuanyan injection + CT	CT	14 d	①②
Wang, (2016)	42/42	28/13	26/15	69 ± 12	71 ± 14	5.3	5	Danshenduofensuanyan injection + CT	CT	14 d	①②
Song et al. (2017)	42/31	-	-	63.01 ± 9.25	63.75 ± 11.83	—	—	Danshenduofensuanyan injection + CT	CT	14 d	①②
Fan et al. (2019)	48/48	25/23	27/21	62.59 ± 8.24	63.21 ± 8.50	4.82 ± 1.79	5.16 ± 1.84	Danshenduofensuanyan injection + CT	CT	2w	②③
Lei and Zhao, (2021)	100/100	61/39	64/36	58.53 ± 8.78	57.62 ± 8.72	4.45 ± 1.38	4.37 ± 1.32	Danshenduofensuanyan injection + CT	CT	2w	①③
Xie, (2018)	80/80	38/42	36/44	67.1 ± 3.7	67.8 ± 4.3	—	—	Danshenduofensuanyan injection + CT	CT	14 d	①
Qi and Guo, (2013)	35/35	21/14	19/16	56–70	55–68	0.58–10	0.67–9	Danshenduofensuanyan injection + CT	CT	2w	①⑦
Yang et al. (2010)	22/20	12/10	12/8	71.56 ± 8.25	70.18 ± 8.26	—	—	Danshenduofensuanyan injection + CT	CT	14 d	①⑦
Xu, (2016)	50/40	29/21	26/14	53.2 ± 4.4	51.3 ± 6.2	—	—	Danshenduofensuanyan injection + CT	CT	10 d	①
Duan, (2016)	44/44	20/24	23/21	71.55 ± 9.35	71.50 ± 10.50	5.25 ± 4.67	5.33 ± 4.67	Danshenduofensuanyan injection + CT	CT	2w	①③
Zhang and Li, (2017)	65/55	38/27	30/25	79.33	79.85	—	—	Danshenduofensuanyan injection + CT	CT	2w	①
Wang et al. (2016)	30/30	15/15	16/14	80	78	—	—	Danshenduofensuanyan injection + CT	CT	14 d	①②⑦
Zhai and Jiao, (2013)	56/56	38/18	37/19	50.4 ± 8.9	51.3 ± 7.8	—	—	Danshenduofensuanyan injection + CT	CT	14 d	①②③
Wang, (2017b)	105/105	63/42	69/36	48.7 ± 11.1	48.4 ± 11.5	4.2 ± 1.9	4.5 ± 1.6	Danshenduofensuanyan injection + CT	CT	2w	①

M, male; F, female; E, experimental group; C, control; CT, conventional treament. ① Total effective rate; ② ECG, efficacy; ③ Angina pectoris attacks; ④ hs-CRP; ⑤ Total cholesterol; ⑥ Triglyceride; ⑦ Adverse events.

### 3.2 Inclusion of studies quality evaluation

**FIGURE 2:**
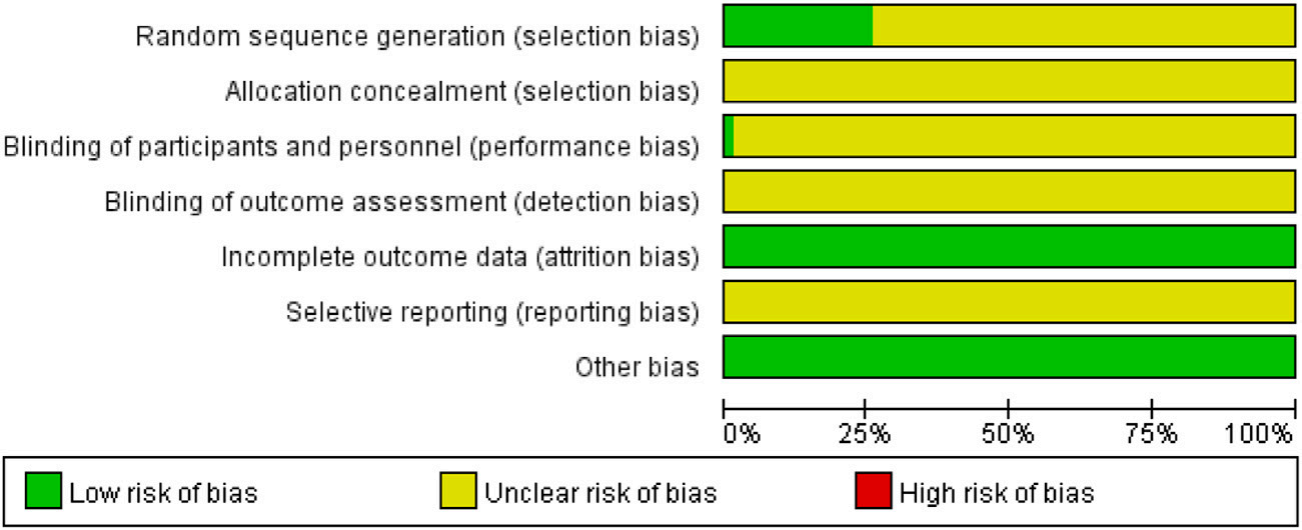
Results of risk of bias evaluation of included studies.

A detailed assessment of the risk of bias for the 148 RCTs studies (Ren, 2017; Li, 2006; Wang and Wu, 2013; Qian et al., 2000; Chen, 2007b; Zhao, 2013; Wang and Guo, 2009; Huang, 2014a; Zhou, 2010; Wu, 2019; Li, 2013; Yang and Ma, 2008; Tian and Wu, 2006; Hao and Wang, 2013; Li and Zhu, 2012; Ma and Peng, 2018; Tang, 2015; Song, 2014; Meng et al., 2014; Wang et al., 2011; Reng et al., 2012; Yu, 2017; Zhang and Lu, 2010; Xu and Hao, 2022; Cai et al., 2014; Liu et al., 2015a; Li et al., 2018; Zhang et al., 2019; Jiang, 2019; Ren et al., 2018; Jiang et al., 2014a; Yang et al., 2014b; Li, 2014a; Gu, 2019; Lan, 2015; Cai et al., 2013; Li, 2018a; Huang, 2017; Hu and Jia, 2014; Zhang, 2017; Yao, 2015; Li and Zhang, 2018; Yu, 2018; Zhao et al., 2015; Huang, 2015; Ma, 2015a; Peng et al., 2015; Yang et al., 2016; Pu, 2017; Wang, 2018; Pi et al., 2019; Lv, 2016; Pu, 2014; Dai, 2015; Chang, 2021; Yang, 2019; Duan, 2019; Liu and Zhang, 2011; Xia, 2011; Pu et al., 2010; Liu, 2015b; Yu and Xu, 2021; Jin and Han, 2020; Zhang HL, 2015; Liang and Dong, 2014; Huang, 2014b; Wang, 2012; An, 2010; Pan, 2012; Lu and Liang, 2015; Yang, 2017a; Liu, 2016; Kang and Hou, 2015; Li, 2018b; Li, 2014b; Li and Lin, 2014; Wang and He, 2011; Xu and Hu, 2016; Zhang, 2016; Jiang et al., 2014b; Huang et al., 2013; Zhao et al., 2012; Du, 2018; Li et al., 2016; Han and Wang, 2011; Chen, 2014; Fang et al., 2016; Guo, 2021; Hu, 2010; Xi et al., 2015; Li and Zhou, 2015; He, 2012; Qin et al., 2010; Chen, 2012; Liang et al., 2011; Luo and Kang, 2011; Qi and Wang, 2011; Liu and Xiao, 2014; Wu et al., 2016; Mao et al., 2016; Chen et al., 2016; Chen et al., 2014; Ren et al., 2012; Han et al., 2016; Liu, 2017; Xia et al., 2016; Zhao et al., 2018; Xu et al., 2013; Li et al., 2014a; Jiao and Wang, 2019; Yldx and As, 2016; Dou, 2010; Liu, 2015c; Yin and Xu, 2013; Qin, 2014; Xu et al., 2011; Kang et al., 2012) is summarized in Figure 2 and Supplementary Figure S1. 1) Selective bias (random sequence generation and allocation concealment): 34 studies (Wang and Guo, 2009; Qin et al., 2010; Chen, 2012; He, 2012; Li, 2013; Wang and Wu, 2013; Huang, 2014b; Chen, 2014; Chen et al., 2014; Gong, 2014; Hu and Jia, 2014; Liu and Xiao, 2014; Pu, 2014; Qin, 2014; Bian, 2015; Lu and Liang, 2015; Fang et al., 2016; Liu, 2016; Mao et al., 2016; Wu et al., 2016; Yldx and As, 2016; Wang, 2017b; Li and Xu, 2017; Ren, 2017; Song et al., 2017; Yu, 2017; Zhang and Li, 2017; Li et al., 2018; Li and Zhang, 2018; Gu, 2019; Pi et al., 2019; Zhang et al., 2019; Guo, 2021; Hao et al., 2021) were randomized by random number table, two studies (Dai, 2015; Zhang, 2016) by dynamic random grouping, one study (Qian et al., 2000) by random parallel grouping, and one study (Liu, 2015b) by “lottery” to determine grouping. Therefore, the risk of selection bias for the above-mentioned studies was considered to be low. The remaining RCTs referred to only random grouping, and the risk of selection bias was considered unclear. 2) Performance bias (blinding of the participants and personnel): Two studies (Kang and Hou, 2015; Jiang, 2019) were single-blind, which were considered low risk. Other studies did not provide information on blinding, so the performance bias was evaluated as unclear risk. 3) Detection bias: All studies did not provide enough information to evaluate its risk level; therefore, the risk is unclear. 4) Attrition bias: None of the included RCTs had incomplete data, so the risk of attrition bias was considered “low.” 5) Reporting bias: Considering that complete implementation protocols were not available for all studies, the risk of reporting bias was considered “unclear”. 6) Other bias: The risk of this bias was considered “low,” because no other obvious bias was observed in all studies.

### 3.3 Results of reticulated meta-analysis

#### 3.3.1 Mesh relationship diagram

**FIGURE 3:**
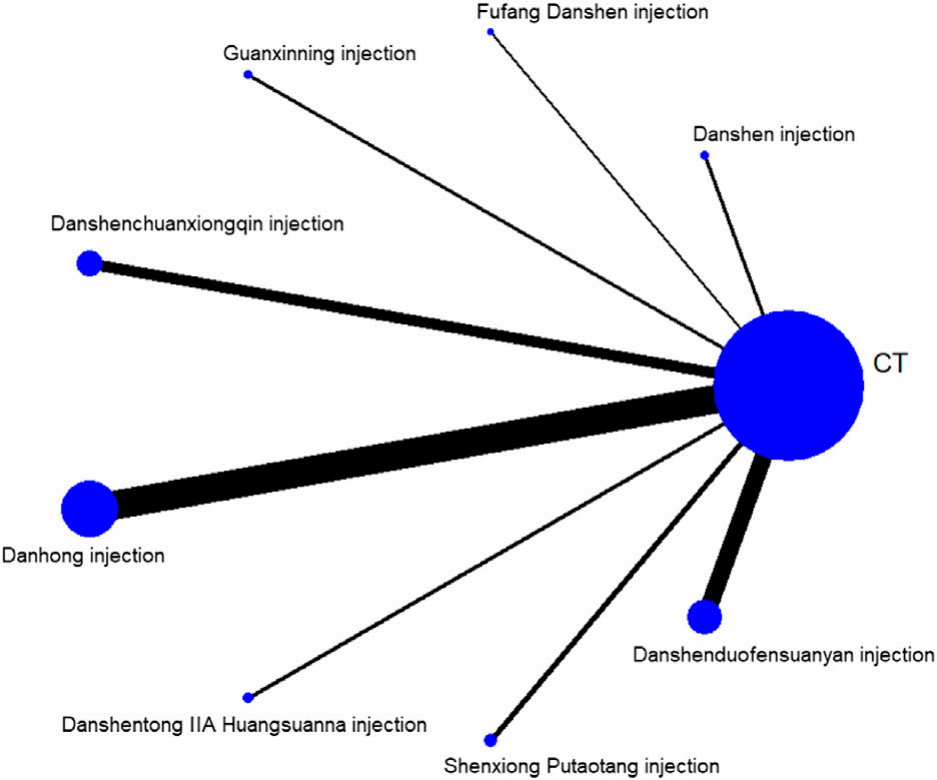
Reticulation of UA treated with SCMI.

The reticulation between the eight included SMIC is shown in Figure 3. The total number of arms in the 148 papers totals 296. Lines between nodes indicate direct comparative evidence between the two interventions, no lines indicate no direct comparison, indirect comparisons can be made through reticulated Meta-analysis. The thickness of the line represents the number of included studies comparing each treatment, and the circular area represents the sample size of the population using the measure.

#### 3.3.2 Consistency testing

All interventions involved in this study did not form a closed loop and did not require consistency testing.

#### 3.3.3 Total effective rate

**FIGURE 4:**
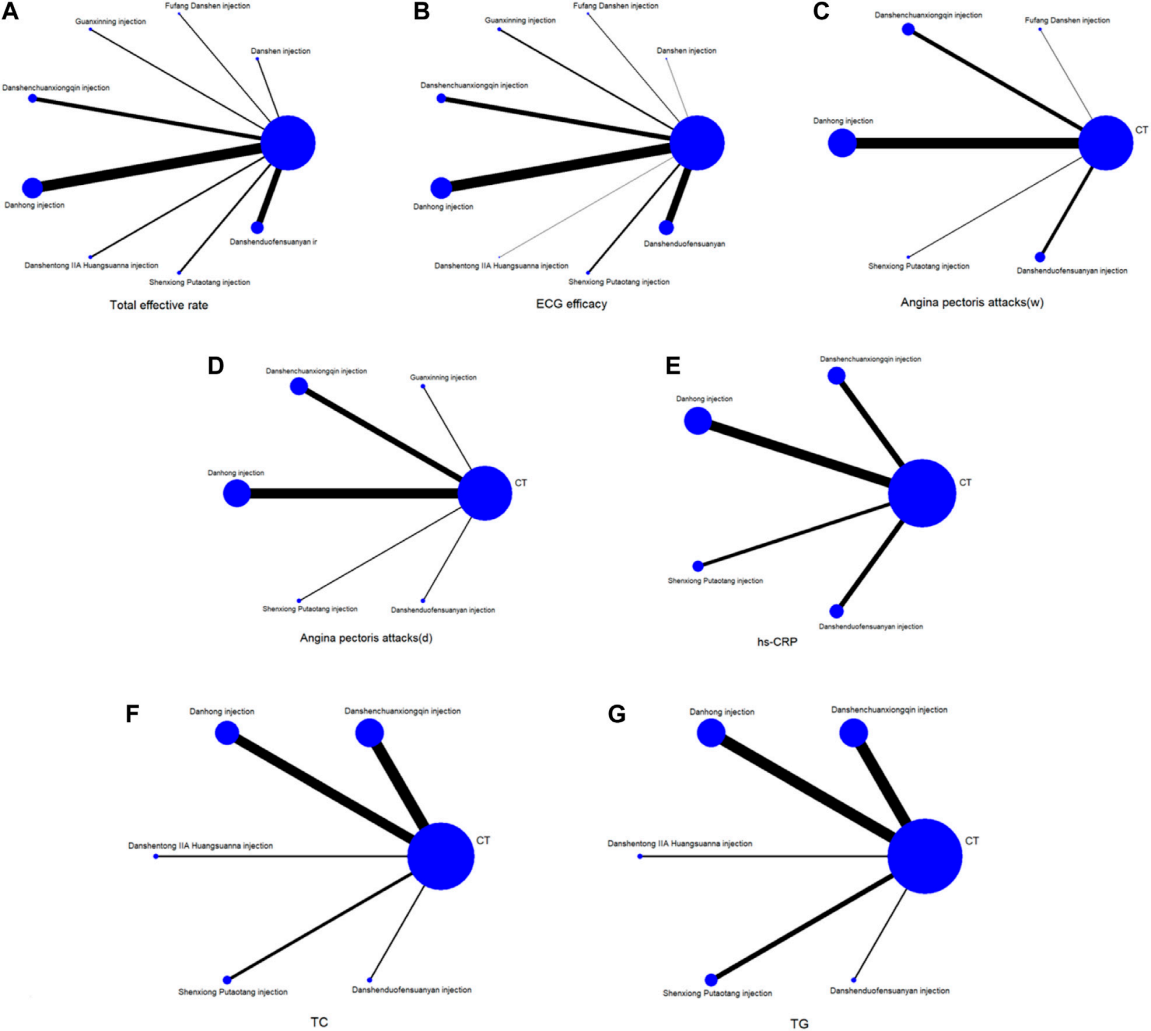
Network diagrams of comparisons on different outcomes of treatments in different groups of patients with UA. **(A)** total effective rate; **(B)** ECG efficacy; **(C)** Angina pectoris attacks (weeks); **(D)** Angina pectoris attacks (days); **(E)** hs-CRP; **(F)** TC; **(G)** TG.

**FIGURE 5:**
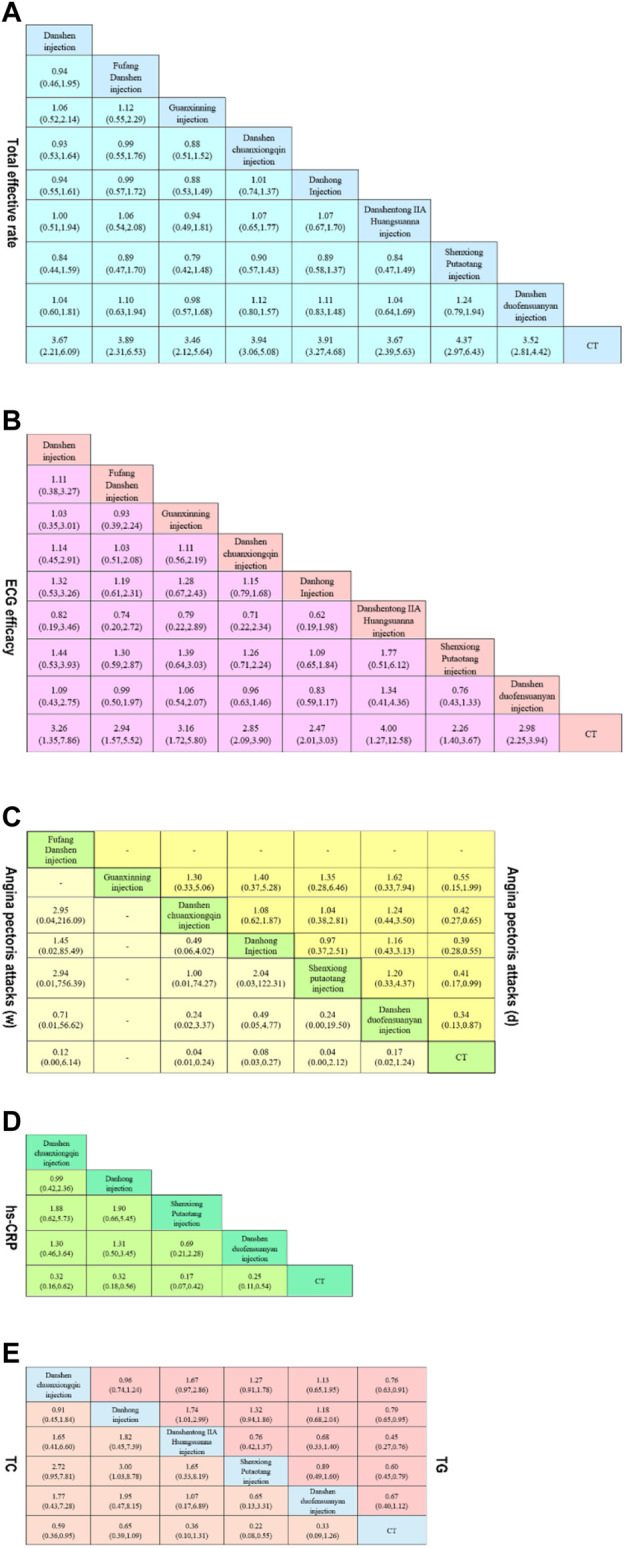
Pooled estimates of the network meta-analysis. **(A)** Pooled risk d ratios (95% credible intervals) for the total effective rate. **(B)** Pooled risk ratios (95% credible intervals) for ECG efficacy. **(C)** Pooled risk ratios (95% credible intervals) Angina pectoris attacks (w and d).**(D)** Pooled risk ratios (95% credible intervals) for hs-CRP.**(E)** Pooled risk ratios (95% credible intervals) for TC and TG.

**FIGURE 6:**
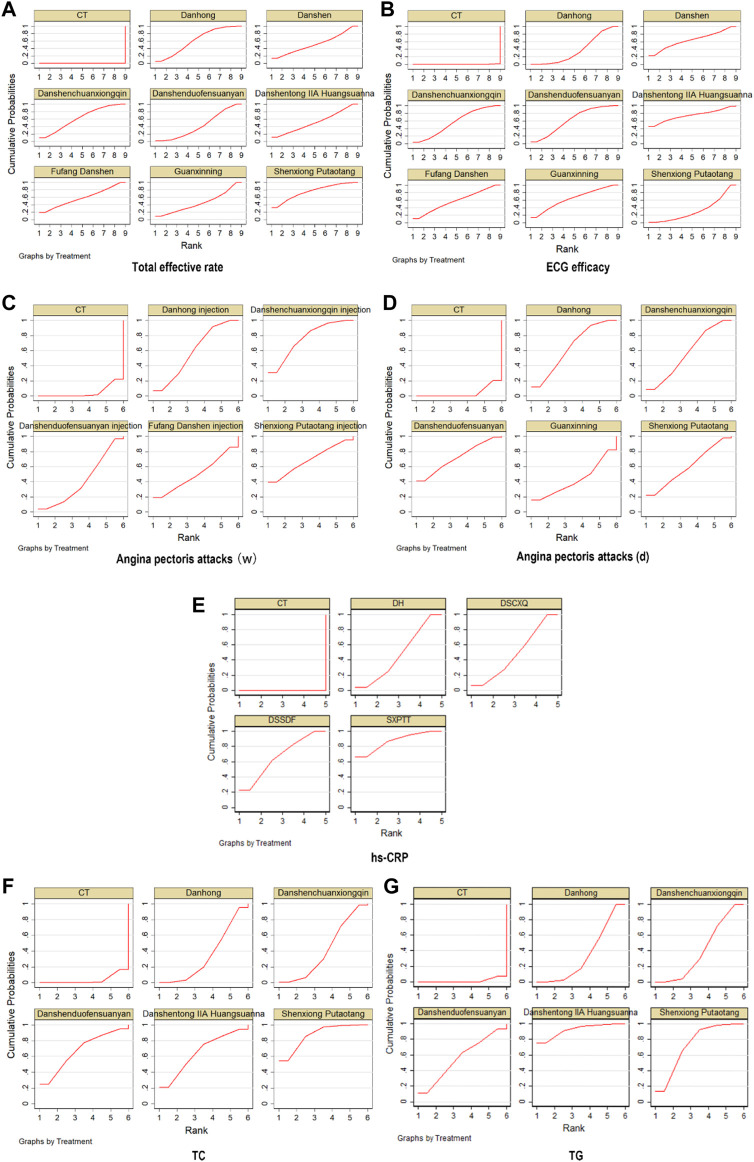
SUCRA of comparisons on different outcomes of treatments in different groups of patients with UA. **(A)** total effective rate; **(B)** ECG efficacy; **(C)** Angina pectoris attacks (weeks); **(D)** Angina pectoris attacks (days); **(E)** hs-CRP; **(F)** TC; **(G)** TG.

A total of 140 studies (Qian et al., 2000; Li, 2006; Tian and Wu, 2006; Chen, 2007b; Yang and Ma, 2008; Wang and Guo, 2009; Zhang and Lu, 2010; Zhou, 2010; Wang et al., 2011; Li and Zhu, 2012; Reng et al., 2012; Hao and Wang, 2013; Li, 2013; Wang and Wu, 2013; Zhao, 2013; Huang, 2014a; Meng et al., 2014; Song, 2014; Tang, 2015; Ren, 2017; Yu, 2017; Ma and Peng, 2018; Wu, 2019; Cai et al., 2013; Jiang et al., 2014a; Li, 2014a; Yang et al., 2014b; Cai et al., 2014; Liu et al., 2015a; Lan, 2015; Li, 2018a; Li et al., 2018; Ren et al., 2018; Gu, 2019; Jiang, 2019; Zhang et al., 2019; Xu and Liu, 2011; Hu and Jia, 2014; Zhang et al., 2014; Ma, 2015a; Bian, 2015; Liu et al., 2015b; Huang, 2015; Huang et al., 2015; Peng et al., 2015; Yao, 2015; Zhao et al., 2015; Zhang, 2017; Li and Zhang, 2018; Yu, 2018; Liu and Zhang, 2011; Xia, 2011; Pu, 2014; Dai, 2015; Lv, 2016; Pu, 2017; Wang, 2018; Duan, 2019; Pi et al., 2019; Yang, 2019; Chang, 2021; Liu, 2015b; Jin and Han, 2020; Zhang HL, 2015; Liang and Dong, 2014; Huang, 2014b; Wang, 2012; An, 2010; Pan, 2012; Lu and Liang, 2015; Yang, 2017a; Liu, 2016; Kang and Hou, 2015; Li, 2018b; Li, 2014b; Li and Lin, 2014; Wang and He, 2011; Xu and Hu, 2016; Zhang, 2016; Jiang et al., 2014b; Xu and Meng, 2008; Xu and Lv, 2017; Chen and Han, 2019; Hao et al., 2021; Gong, 2014; Wang, 2014; Lai, 2012; Ning et al., 2011; Gao et al., 2011; He and Sun, 2012; Lv and Liu, 2011; Ju, 2013; Li and Xu, 2017; Huang et al., 2013; Zhao et al., 2012; Du, 2018; Li et al., 2016; Han and Wang, 2011; Qin et al., 2010; Liang et al., 2011; Luo and Kang, 2011; Qi and Wang, 2011; Chen, 2012; He, 2012; Chen et al., 2014; Liu and Xiao, 2014; Li and Zhou, 2015; Chen et al., 2016; Mao et al., 2016; Wu et al., 2016; Dou, 2010; Xu et al., 2011; Kang et al., 2012; Xu et al., 2013; Yin and Xu, 2013; Li et al., 2014a; Qin, 2014; Liu, 2015c; Han et al., 2016; Wang, 2016; Xia et al., 2016; Yldx and As, 2016; Liu, 2017; Song et al., 2017; Zhao et al., 2018; Jiao and Wang, 2019; Yang et al., 2010; Qi and Guo, 2013; Zhai and Jiao, 2013; Duan, 2016; Wang et al., 2016; Xu, 2016; Wang, 2017b; Zhang and Li, 2017; Xie, 2018; Lei and Zhao, 2021) were included to compare the total effective rate after SMIC treatment. The evidence diagram is shown in Figure 4A. Danshen injection, Fufang Danshen injection, Guanxinning injection, Danshenchuanxiongqin injection, Danhong injection, Danshentong IIA Huangsuanna injection, Shenxiong Putaotang injection and Danshenduofensuanyan injection were added respectively compared with CT, for Total effective rate the difference was statistically significant (*p* < 0.05). See Figure 5A. The eight SMIC total effective rate in descending order (rank) are:Shenxiong Putaotang injection (SUCRA = 75.2%) > Danshenchuanxiongqin injection (SUCRA = 62.6%) > Danhong injection (SUCRA = 61.7%) > Fufang Danshen injection (SUCRA = 58.5%) > Danshen injection (SUCRA = 52.1%) > Danshentong IIA Huangsuanna injection (SUCRA = 51.9%) > Guanxinning injection (SUCRA = 45.3%) > Danshenduofensuanyan injection (SUCRA = 42.6%) > CT (SUCRA = 0.0%). The probability ranking is shown in Figure 6A.

#### 3.3.4 ECG efficacy

A total of 61 studies (Li, 2006; Yang and Ma, 2008; Wang and Guo, 2009; Zhang and Lu, 2010; Zhou, 2010; Li and Zhu, 2012; Cai et al., 2013; Jiang et al., 2014a; Li, 2014a; Meng et al., 2014; Song, 2014; Zhang et al., 2014; Liu et al., 2015a; Bian, 2015; Lan, 2015; Tang, 2015; Li et al., 2018; Ren et al., 2018; Zhang et al., 2019; Xu and Meng, 2008; Pu et al., 2010; Gao et al., 2011; Liu and Zhang, 2011; Lv and Liu, 2011; Wang and He, 2011; Xia, 2011; He and Sun, 2012; Ju, 2013; Jiang et al., 2014b; Gong, 2014; Li and Lin, 2014; Wang, 2014; Kang and Hou, 2015; Lu and Liang, 2015; Zhang HL, 2015; Xu and Hu, 2016; Yang et al., 2016; Yang, 2017a; Pu, 2017; Wang, 2018; Pi et al., 2019; Dou, 2010; Qin et al., 2010; Luo and Kang, 2011; Qi and Wang, 2011; Xu et al., 2011; Chen, 2012; Kang et al., 2012; Xu et al., 2013; Yin and Xu, 2013; Zhai and Jiao, 2013; Li et al., 2014a; Chen et al., 2014; Liu and Xiao, 2014; Qin, 2014; Wang, 2016; Wang et al., 2016; Xia et al., 2016; Yldx and As, 2016; Song et al., 2017; Fan et al., 2019) comparing ECG efficacy after SMIC treatment were included. The evidence plots are shown in Figure 4B. The results of the reticulated Meta-analysis showed that the addition of Danshen injection, Fufang Danshen injection, Guanxinning injection, Danshenchuanxiongqin injection, Danhong injection, Danshentong IIA Huangsuanna injection, Shenxiong Putaotang injection, Danshenduofensuanyan injection were added respectively compared with CT, which was statistically significant for ECG efficacy (*p* < 0.05). Figure 5B. The eight SMIC ECG efficacy in order from highest to lowest specific ranking (rank) are:Danshentong IIA Huangsuanna injection (SUCRA = 74.4%) > Danshen injection (SUCRA = 64.2%) > Guanxinning injection (SUCRA = 63.9%) > Danshenduofensuanyan injection (SUCRA = 62.2%) > Fufang Danshen injection (SUCRA = 57.9%) > Danshenchuanxiongqin injection (SUCRA = 56.4%) > Danhong injection (SUCRA = 37.6%) > Shenxiong Putaotang injection (SUCRA = 33.2%) > CT (SUCRA = 0.2%). The probability ranking is shown in Figure 6B.

#### 3.3.5 Angina pectoris attacks

23 studies (Liu and Zhang, 2011; Lv and Liu, 2011; Ning et al., 2011; Lai, 2012; Wang, 2012; Zhai and Jiao, 2013; Huang, 2014a; Li, 2014b; Hu and Jia, 2014; Zhang HL, 2015; Duan, 2016; Liu, 2016; Lv, 2016; Xia et al., 2016; Zhang, 2016; Li and Zhang, 2018; Ren et al., 2018; Chen and Han, 2019; Zhang et al., 2019; Guo, 2021; Lei and Zhao, 2021; Xu and Hao, 2022) with weeks as the unit of time observation and 16 studies (Tian and Wu, 2006; Pu et al., 2010; Zhang and Lu, 2010; Xu and Liu, 2011; Yang et al., 2014b; Pu, 2014; Wang, 2014; Liu, 2015b; Dai, 2015; Huang, 2017; Yu, 2017; Zhang, 2017; Duan, 2019; Jiao and Wang, 2019; Chang, 2021) with days as the unit of time observation were included, respectively. The evidence plots are shown in Figures 4C,D, respectively. The results of the NMA showed that there was no statistically significant (*p* > 0.05) change in angina episodes with CT versus the addition of SMIC, with weeks as the time observation unit or with days as the time observation unit. See Figure 5C. The five SMIC were ranked from highest to lowest in order of improvement of Angina pectoris attacks, using weeks as the unit of observation time, as follows:Danshenchuanxiongqin injection (SUCRA = 76.0%) > Shenxiong Putaotang injection (SUCRA = 69.0%) > Danhong injection (SUCRA = 58.7%) > Fufang Danshen injection (SUCRA = 49.9%) > Danshenduofensuanyan injection (SUCRA = 41.6%) > CT (SUCRA = 4.8%). The probability ranking is shown in Figure 6C. The five SMIC were ranked in order of improvement of Angina pectoris attacks from highest to lowest, using days as the unit of time observation: Danshenduofensuanyan injection (SUCRA = 72.2%) > Danhong injection (SUCRA = 63.9%) > Shenxiong Putaotang injection (SUCRA = 60.1%) > Danshenchuanxiongqin injection (SUCRA = 57.0%) > Guanxinning injection (SUCRA = 42.6%) > CT (SUCRA = 4.3%). The probability ranking is shown in Figure 6D.

#### 3.3.6 hs-CRP

A total of 20 studies (Hu, 2010; Gao et al., 2011; He, 2012; Qi and Guo, 2013; Cai et al., 2014; Chen et al., 2014; Liu and Xiao, 2014; Liu et al., 2015a; Ma, 2015a; Liu et al., 2015b; Kang and Hou, 2015; Peng et al., 2015; Xi et al., 2015; Zhao et al., 2015; Duan, 2016; Lv, 2016; Mao et al., 2016; Wu et al., 2016; Pu, 2017; Li et al., 2018; Xie, 2018; Gu, 2019; Jiang, 2019) comparing hs-CRP changes after SMIC treatment were included. The evidence plots are shown in Figure 4E. The results of NMA showed that the addition of Danshenchuanxiongqin injection, Danhong injection, Shenxiong Putaotang injection and Danshenduofensuanyan injection was statistically significant (*p* < 0.05) for the improvement of hs-CRP compared with CT. See Figure 5D. The specific ranking (rank) of the four SMIC in order of highest to lowest probability of improving hs-CRP was:Shenxiong Putaotang injection (SUCRA = 87.1%) > Danshenduofensuanyan injection (SUCRA = 66.5%) > Danshenchuanxiongqin injection (SUCRA = 48.9%) > Danhong injection (SUCRA = 47.5%) > CT (SUCRA = 0.0%). The probability ranking is shown in Figure 6E.

#### 3.3.7 TC and TG

A total of 17 studies (An, 2010; Wang et al., 2011; He, 2012; Pan, 2012; Reng et al., 2012; Cai et al., 2014; Chen, 2014; Hu and Jia, 2014; Liu et al., 2015a; Xi et al., 2015; Yao, 2015; Han et al., 2016; Pu, 2017; Wang, 2018; Chen and Han, 2019; Zhang et al., 2019; Yu and Xu, 2021) comparing changes in TC after SMIC treatment; 19 studies (An, 2010; Hu, 2010; Wang et al., 2011; He, 2012; Pan, 2012; Reng et al., 2012; Cai et al., 2014; Chen, 2014; Hu and Jia, 2014; Liu et al., 2015a; Xi et al., 2015; Yao, 2015; Han et al., 2016; Lv, 2016; Pu, 2017; Wang, 2018; Chen and Han, 2019; Zhang et al., 2019; Yu and Xu, 2021) compar-ing changes in TG after SMIC treatment were included. The evidence plots are shown in Figures 4F,G. The results of the reticulated Meta-analysis showed that in terms of TC improvement, the addition of Danshenchuanxiongqin injection had MD = 0.59, 95% CI [0.36, 0.95] compared with CT; the addition of Shenxiong Putaotang injection had MD = 0.22, 95% CI [0.08, 0.55], which was statistically significant for the improvement of TC (*p* < 0.05). See Figure 5E. The specific ranking (rank) of the five SMIC in order of probability of improving TC is: Shenxiong Putaotang injection (SUCRA = 87.3%) > Danshenduofensuanyan injection (SUCRA = 67.8%) > Danshentong IIA Huangsuanna injection (SUCRA = 65.2%) > Danshenchuanxiongqin injection (SUCRA = 41.5%) > Danhong injection (SUCRA = 34.7%) > CT (SUCRA = 3.6%). The probability ranking is shown in Figure 6F.

The addition of Danshenchuanxiongqin injection, Danhong injection, Danshentong IIA Huangsuanna injection, Shenxiong Putaotang injection, and Danshenduofensuanyan injection was statistically significant (*p* < 0.05) for the improvement of TG. See Figure 5E. The specific ranking (rank) of the five SMIC in order of their probability of improving TG is: Danshentong IIA Huangsuanna injection (SUCRA = 92.4%) > Shenxiong Putaotang injection (SUCRA = 73.9%) > Danshenduofensuanyan injection (SUCRA = 56.1%) > Danshenchuanxiongqin injection (SUCRA = 41.4%) > Danhong injection (SUCRA = 34.8%) > CT (SUCRA = 1.4%). The probability ranking is shown in Figure 6G.

#### 3.3.8 Cluster analysis and meta-analysis two-by-two comparison results

**FIGURE 7:**
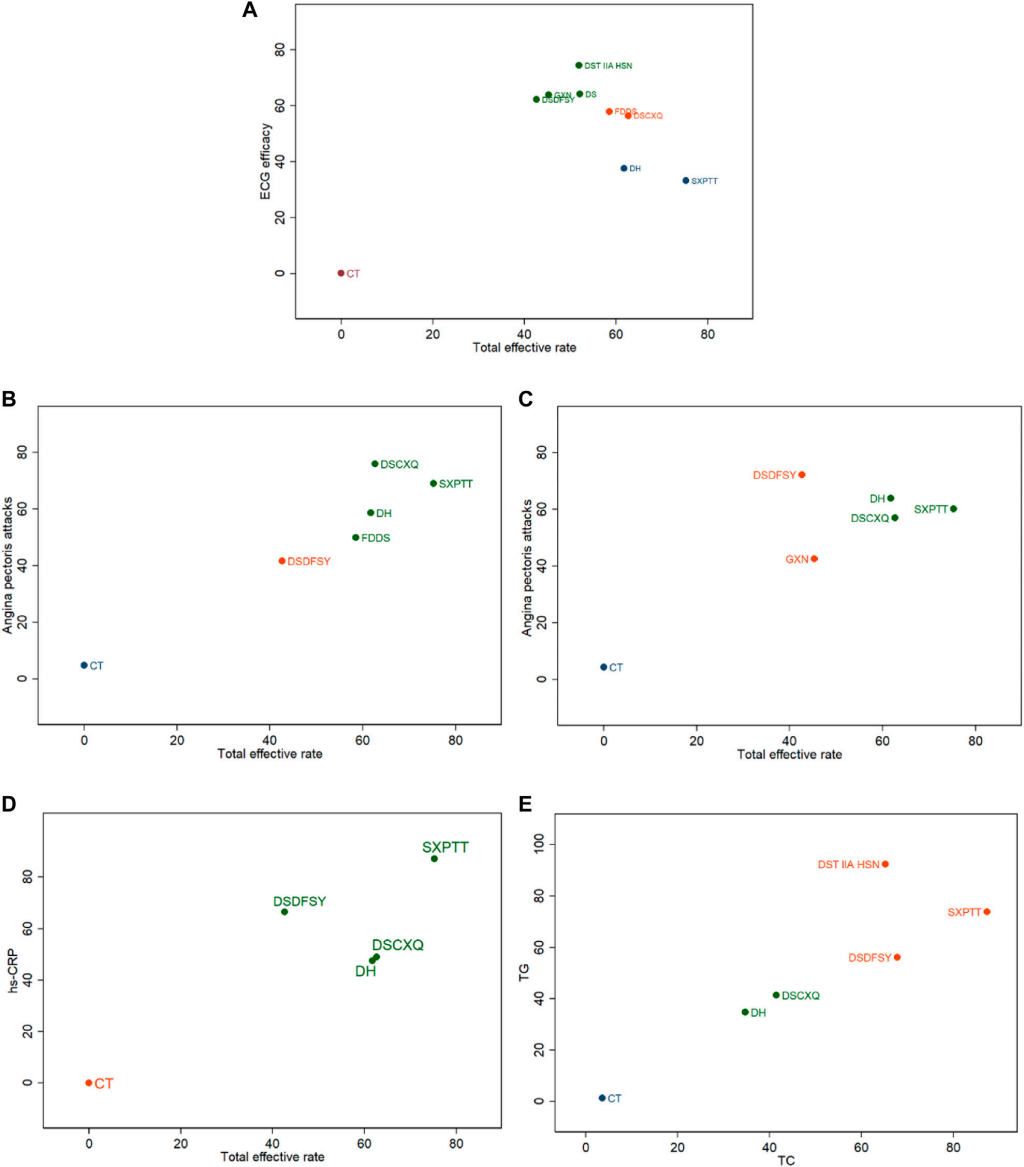
Cluster analysis of comparisons on different outcomes of treatments in different groups of patients with UA. **(A)** total effective rate and ECG efficacy; **(B)** total effective rate and Angina pectoris attacks (weeks); **(C)** total effective rate and Angina pectoris attacks (days); **(D)** total effective rate and hs-CRP; **(E)** TC and TG.

The key outcome indicators included in this study were cluster analyzed to derive the intervention of different SMIC for two outcome indicators at the same time. Total effective rate was clustered with ECG efficacy, with Angina pectoris attacks, and with hs-CRP, respectively, and the results are shown in Figures 7A–D; two lipid indicators were clustered to assess the improvement of SMIC on TC and TG, and the results are shown in Figure 7E. In terms of total effective rate versus ECG efficacy, Danshentong IIA Huangsuanna injection, Guanxinning injection and Danshen injection in the upper right corner of Figure 7A performed better; in terms of total effective rate and Angina pectoris attacks, Danshenchuanxiongqin injection and Shenxiong Putaotang injection performed better with weeks as the unit of observation, while with days as the unit of observation, the Danshenduofensuanyan injection and Danhong injection performed better in terms of total effective rate and hs-CRP improvement; Shenxiong Putaotang injection in the upper right corner of Figure 7D performed better; for TC and TG for the improvement of TC and TG, Danshentong IIA Huangsuanna injection and Shenxiong Putaotang injection performed better.

The eight SMIC included in the study were compared two-by-two under each outcome indicator (Supplementary Figure S2). It can be concluded that in terms of Total effective rate, all eight SMIC are better than CT; Danshenchuanxiongqin injection and Danhong injection are superior to CT in ECG efficiency; In angina pastoris attacks, with week as the observation index, danshenchuanxiongqin injection and Danhong injection are better than CT, and with day as the observation index, danshenchuanxiongqin injection, Danhong injection, Shenxiong putaotang injection and danshenduofensanyan injection are better than CT; In terms of hs-CRP improvement, danshenchuanxiongqin injection, Danhong injection, Shenxiong putaotang injection and danshenduofensanyan injection are better than CT; In terms of TC, TG, Shenxiong Putaotang injection, Danshenchuanxiongqin injection, Danhong injection, Danshentong IIA Huangsuanna injection are better than CT.

#### 3.3.9 Sensitivity analysis

Of the included SMIC, 99 studies (Qian et al., 2000; Tian and Wu, 2006; Chen, 2007b; Yang and Ma, 2008; Wang et al., 2011; Xu and Liu, 2011; Reng et al., 2012; Cai et al., 2013; Zhao, 2013; Jiang et al., 2014a; Hu and Jia, 2014; Zhang et al., 2014; Bian, 2015; Lan, 2015; Tang, 2015; Huang, 2017; Yu, 2017; Zhang, 2017; Li, 2018a; Ma and Peng, 2018; Ren et al., 2018; Yu, 2018; Wu, 2019; An, 2010; Pu et al., 2010; Gao et al., 2011; Liu and Zhang, 2011; Lv and Liu, 2011; Wang and He, 2011; Lai, 2012; Pan, 2012; Huang, 2014b; Jiang et al., 2014b; Gong, 2014; Li and Lin, 2014; Liang and Dong, 2014; Pu, 2014; Wang, 2014; Ma, 2015a; Liu, 2015b; Liu et al., 2015b; Dai, 2015; Kang and Hou, 2015; Lu and Liang, 2015; Peng et al., 2015; Zhao et al., 2015; Liu, 2016; Xu and Hu, 2016; Zhang, 2016; Pu, 2017; Xu and Lv, 2017; Wang, 2018; Duan, 2019; Pi et al., 2019; Jin and Han, 2020; Hao et al., 2021; Yu and Xu, 2021; Huang et al., 2013; Zhao et al., 2012; Du, 2018; Li et al., 2016; Han and Wang, 2011; Chen, 2014; Fang et al., 2016; Hu, 2010; He, 2012; Qin et al., 2010; Chen, 2012; Liang et al., 2011; Luo and Kang, 2011; Mao et al., 2016; Chen et al., 2016; Chen et al., 2014; Ren et al., 2012; Han et al., 2016; Liu, 2017; Xia et al., 2016; Xu et al., 2013; Li et al., 2014a; Jiao and Wang, 2019; Yldx and As, 2016; Dou, 2010; Liu, 2015c; Yin and Xu, 2013; Qin, 2014; Xu et al., 2011; Kang et al., 2012; Wang, 2016; Song et al., 2017; Fan et al., 2019; Lei and Zhao, 2021; Xie, 2018; Qi and Guo, 2013; Yang et al., 2010) with an intervention duration of 2 weeks (14 days), for a total of 10,246 patients, were included in the sensitivity analysis. The results found that Total effective rate, ECG efficacy, and hs-CRP did not show any significant deviation from the original NMA; the addition of Danshenduofensuanyan injection was more effective compared with CT in terms of angina efficacy, using w as the time measurement unit. Using TC as the observation, the addition of Danshentong IIA Huangsuanna injection or Danshenduofensuanyan injection was more effective compared with CT, while using TG as the observation, the addition of Danshenchuanxiongqin injection or Danhong injection was opposite to the original NMA (Supplementary Figures S3).

A sensitivity analysis was performed conditional on the number of cases ≥90, and a total of 79 studies (Li, 2006; Yang and Ma, 2008; Zhou, 2010; Wang et al., 2011; Reng et al., 2012; Cai et al., 2013; Li, 2013; Jiang et al., 2014a; Yang et al., 2014b; Cai et al., 2014; Meng et al., 2014; Zhang et al., 2014; Liu et al., 2015a; Bian, 2015; Lan, 2015; Yao, 2015; Huang, 2017; Ren, 2017; Li, 2018a; Li and Zhang, 2018; Ma and Peng, 2018; Ren et al., 2018; Gu, 2019; Wu, 2019; Zhang et al., 2019; Gao et al., 2011; Liu and Zhang, 2011; Wang and He, 2011; He and Sun, 2012; Lai, 2012; Pan, 2012; Huang, 2014b; Li, 2014b; Gong, 2014; Liang and Dong, 2014; Liu, 2015b; Liu et al., 2015b; Huang, 2015; Kang and Hou, 2015; Lu and Liang, 2015; Peng et al., 2015; Zhang HL, 2015; Zhao et al., 2015; Liu, 2016; Lv, 2016; Xu and Hu, 2016; Zhang, 2016; Yang, 2017a; Li and Xu, 2017; Xu and Lv, 2017; Chen and Han, 2019; Duan, 2019; Pi et al., 2019; Yang, 2019; Hao et al., 2021; Dou, 2010; Qin et al., 2010; Han and Wang, 2011; Luo and Kang, 2011; Xu et al., 2011; Xu et al., 2013; Yin and Xu, 2013; Zhai and Jiao, 2013; Li et al., 2014a; Chen, 2014; Liu and Xiao, 2014; Li and Zhou, 2015; Chen et al., 2016; Li et al., 2016; Mao et al., 2016; Wu et al., 2016; Xu, 2016; Wang, 2017b; Zhang and Li, 2017; Du, 2018; Xie, 2018; Zhao et al., 2018; Fan et al., 2019; Lei and Zhao, 2021) with 10,013 patients were included. In angina pectoris efficacy (weeks), the addition of Danshenduofensuanyan injection was different from the original results; regarding angina pectoris efficacy (days) the treatment effect of adding Shenxiong Putaotang injection versus adding Danshenduofensuanyan injection were different from the original results; the improvement of hs-CRP and TC with the addition of Danshenchuanxiongqin injection was different from the original results; and with regard to the improvement of TG, the results with the addition of Danhong injection were different from the original results (Supplementary Figure S4). The effect of the number of cases on the study results was more pronounced, and when studies with ≥90 cases were analyzed, most observations yielded different results than before, suggesting that for more robust results, the number of patients involved in the study needs to be planned rationally to make the study more convincing.

#### 3.3.10 The small sample effect and publication bias

**FIGURE 8:**
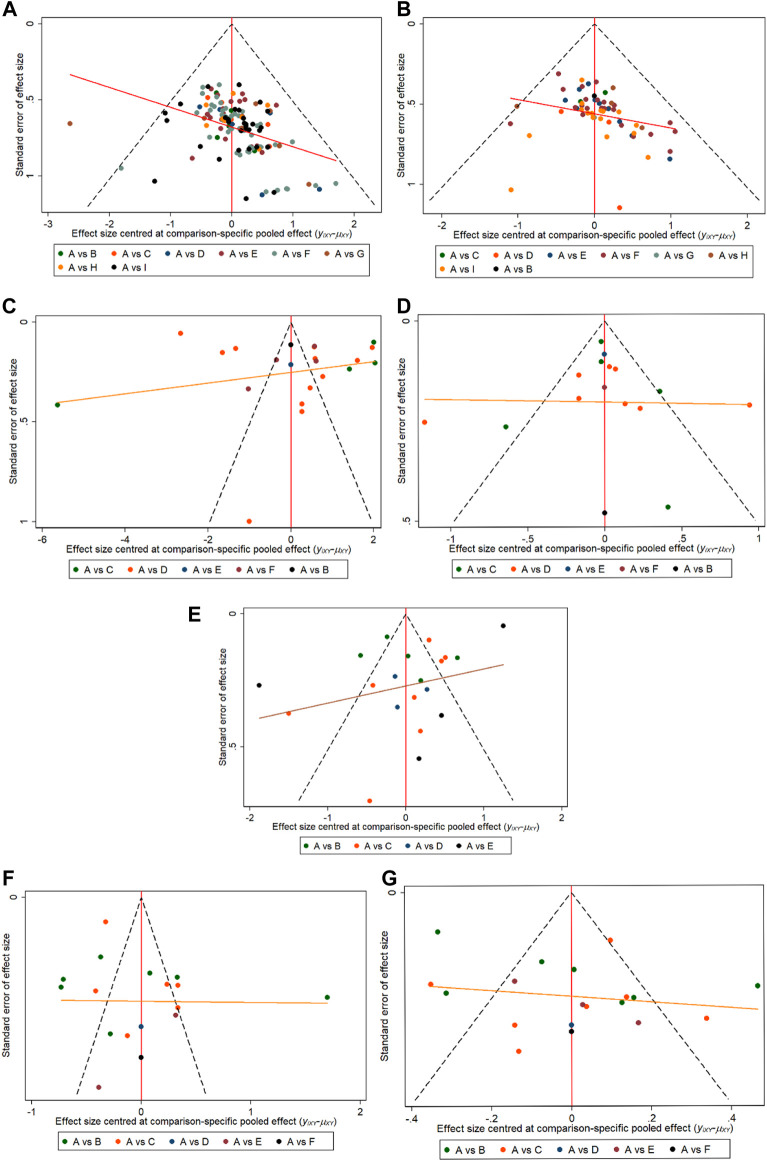
Comparison-correction funnel chart for ending indicator, **(A)** Inclusion of total effective rate; **(B)** ECG efficacy; **(C)** Angina pectoris attack (weeks); **(D)** Angina pectoris attack (days); **(E)** hs-CRP; **(F)** TC; **(G)** TG.

Inclusion of total effective rate (Significantly effective + Effective); ECG efficacy (Significantly effective + Effective); Angina pectoris attack; hs- CRP (High sensitivity C-reactive protein); TC (total cholesterol); and TG (triglyceride) as evaluation metrics to produce comparative corrected funnel plots for studies to assess small sample effects, see Figure 8. The results showed that total effective rate, ECG efficacy and hs-CRP comparative correction funnel plot showed basic symmetry and the studies were roughly symmetrically distributed on both sides of the midline, suggesting a small sample effect is less likely. angina pectoris attack (including weeks as observation unit and days as observation unit), TC and TG comparison-corrected funnel plot symmetry was poor, suggesting the possibility of a small sample effect. The reasons may be related to differences in the quality of included studies, small sample size, inconsistent treatment regimens, and different types of herbal injections used and intervention regimens.(A: CT, B: Danshen injection, C: Fufang Danshen injection, D: Guanxinning injection, E: Danshenchuanxiongqin injection, F: Danhong injection, G: Danshentong IIA Huangsuanna injection, H: Shenxiong Putaotang injection, I: Danshenduofensuanyan injection).

### 3.4 Adverse reactions

A total of 63 studies (Tian and Wu, 2006; Wang and Guo, 2009; Zhang and Lu, 2010; Zhou, 2010; Reng et al., 2012; Cai et al., 2013; Hao and Wang, 2013; Wang and Wu, 2013; Zhao, 2013; Jiang et al., 2014a; Yang et al., 2014b; Zhang et al., 2014; Bian, 2015; Huang et al., 2015; Lan, 2015; Huang, 2017; Ren, 2017; Yu, 2017; Li and Zhang, 2018; Ma and Peng, 2018; Wu, 2019; Xu and Meng, 2008; Lv and Liu, 2011; Ning et al., 2011; Wang and He, 2011; He and Sun, 2012; Wang, 2012; Ju, 2013; Huang, 2014b; Jiang et al., 2014b; Li and Lin, 2014; Wang, 2014; Lu and Liang, 2015; Zhang HL, 2015; Liu, 2016; Lv, 2016; Xu and Hu, 2016; Yang et al., 2016; Zhang, 2016; Li, 2018b; Hao et al., 2021; Yu and Xu, 2021; Qin et al., 2010; Yang et al., 2010; Liang et al., 2011; Chen, 2012; Ren et al., 2012; Zhao et al., 2012; Qi and Guo, 2013; Xu et al., 2013; Yin and Xu, 2013; Li et al., 2014a; Chen, 2014; Chen et al., 2014; Qin, 2014; Wang et al., 2016; Xia et al., 2016; Liu, 2017; Xie, 2018; Jiao and Wang, 2019) mentioned adverse reactions as one of the observations during the study, of which 38 studies (Tian and Wu, 2006; Xu and Meng, 2008; Zhang and Lu, 2010; Lv and Liu, 2011; Ning et al., 2011; He and Sun, 2012; Reng et al., 2012; Wang, 2012; Cai et al., 2013; Wang and Wu, 2013; Zhao, 2013; Huang, 2014b; Jiang et al., 2014b; Yang et al., 2014b; Cai et al., 2014; Li and Lin, 2014; Zhang et al., 2014; Liu et al., 2015a; Bian, 2015; Lu and Liang, 2015; Zhang HL, 2015; Xu and Hu, 2016; Yang et al., 2016; Zhang, 2016; Li, 2018b; Hao et al., 2021; Yang et al., 2010; Han and Wang, 2011; Ren et al., 2012; Zhao et al., 2012; Qi and Guo, 2013; Xu et al., 2013; Yin and Xu, 2013; Li et al., 2014a; Qin, 2014; Li et al., 2016; Xia et al., 2016; Liu, 2017; Xie, 2018) did not observe significant adverse reactions and 24 studies (Yang and Ma, 2008; Wang and Guo, 2009; Qin et al., 2010; Zhou, 2010; Wang and He, 2011; Ju, 2013; Jiang et al., 2014a; Chen, 2014; Chen et al., 2014; Hu and Jia, 2014; Wang, 2014; Huang et al., 2015; Lan, 2015; Liu, 2016; Lv, 2016; Wang et al., 2016; Huang, 2017; Ren, 2017; Yu, 2017; Li and Zhang, 2018; Ma and Peng, 2018; Jiao and Wang, 2019; Wu, 2019; Yu and Xu, 2021) recorded significant adverse reactions, the results are shown in Table 2.

**TABLE 2:** Occurrence of adverse reactions of SMIC.

Injections	Sample size	Headache	Dizziness	Nausea	Vomit	Abdominal distention	Palpitation	Insomnia	Rash	Flush face	Unspecified
Danshen injection	48	1	0	0	0	0	0	1	2	0	0
Fufang Danshen injection	189	1	2	0	0	0	0	0	1	2	0
Guanxinning injection	89	5	0	0	0	0	0	0	0	0	3
Danshen chuanxiongqin injection	325	1	0	4	3	2	0	0	8	0	0
Danhong injection	355	1	1	11	0	4	3	0	2	0	1
Danshentong IIA Huangsuanna injection	116	1	0	0	0	0	0	0	0	5	0
Shenxiong Putaotang injection	56	0	0	0	0	0	0	0	0	0	0
Danshen duofensuanyan injection	106	2	1	1	0	0	0	0	0	0	0
CT	1,242	19	4	22	3	2	2	1	3	1	10

## 4 Discussion

In this NMA, A comprehensive summary of the efficacy and safety of different SMIC in patients with UA was presented. The results showed that 1) All SMIC combined with CT were superior to CT alone in terms of total efficiency, ECG efficiency, angina attack, hs-CRP, TC, and TG; 2) Shenxiong Putaotang, Danshentong IIA Huangsuanna, Danshenchuanxiongqin (time observation unit in weeks), Danshenduofensuanyan (time observation unit in days), Shenxiong Putaotang, Shenxiong Putaotang and Danshentong IIA Huangsuanna performed best as measured by total efficiency, ECG efficiency, angina attack, hs-CRP, TC, and TG, respectively; 3) Shenxiong Putaotang performed best in terms of overall efficiency, better than other SMIC, but only better than CT in terms of ECG efficacy; 4) Both Shenxiong Putaotang and Danhong performed better than most SMIC in the measure of Angina pectoris attacks; 5) Shenxiong Putaotang and Danshentong IIA Huangsuanna were the two best performing treatments in terms of lipid markers (TC and TG); 6) Shenxiong Putaotang and Danshentong IIA Huangsuanna, which outperformed other SMICs in several study metrics. Combining the results of each index, Shenxiong Putaotang injection and Danshentong IIA Huangsuanna injection have better improvement for major and minor indexes, and can be preferentially selected for patient treatment.

In addition, based on the results, we noted that Shenxiong Putaotang outperformed other SMICs in three indicators (total effective rate, hs-CRP and TC) and ranked second in improving TG, suggesting that Shenxiong Putaotang injection is superior in improving symptoms, slowing inflammatory response and optimizing lipids. Previous studies on Shenxiong Putaotang injection have shown its role in protecting cardiomyocytes and reducing the inflammatory response. The present results suggest that the optimization of lipid levels by Shenxiong Putaotang injection is also one of its benefits, and a deeper mechanism in this direction can be explored. Danshentong IIA Huangsuanna injection performed best in both indices: ECG efficacy and TC. The improvement in ECG may be related to enhanced coronary blood flow, which restores collateral circulation and local blood supply in the ischemic region, while the control of TC needs to be explained by further studies.

In the clinical setting, therapeutic effects and safety are the two aspects of CMIs applications that receive the most attention. The evaluation of safety not only helps to avoid the occurrence of adverse reactions and find solutions to deal with them, but also summarizes the mechanism of the occurrence of adverse reactions. A total of 62 studies in this NMA included adverse reactions as an observation, 24 of which mentioned specific symptoms and the number of people experiencing them, and the remaining studies did not report any serious adverse events (see Table 2). According to recorded adverse reactions, common side effects of SMIC are nausea, headache and rash; occasionally dizziness, abdominal distension, facial flushing, palpitations, insomnia and vomiting are seen. Among the patients using SMIC, a total of 12 patients with headache symptoms and a total of 16 patients with nausea symptoms were observed, both of which were associated with a greater effect on the digestive and circulatory systems of the beneficial qi and blood-boosting injections represented by SMIC (Wang et al., 2019b). There were also a similarly high number of rash symptoms in a total of 13 cases, which is generally consistent with the results reported in the previous literature: most adverse reactions after CMI occurred in the skin (Yuan et al., 2021). The reason may be that CMI contains large molecules (Li, 2018c) (including: peptides, polysaccharides, etc.), which enter the body’s blood circulation through the veins and may cause an immune response, followed by a systemic reaction, which includes skin. It is also reported at a higher rate because adverse skin reactions are the easiest to observe and not easily confused with the original disease (Hong and Ma, 2011).

These symptoms can be effectively relieved by reducing the application, discontinuing the medication, or treating the symptoms. Nevertheless, clinicians should be aware of the possibility of adverse reactions when applying SMIC treatment.

Regarding the causes of adverse reactions to injections, on the one hand, the use of CMIs is not standardized, such as the dose or dosing concentration exceeds the routine, and the drip rate is not strictly controlled; on the other hand, the mixing of CMIs with chemical drugs and other combinations greatly increases the incidence of adverse reactions to CMIs, because the thermogenic and allergic superimposed effects of drugs amplify the incidence of adverse reactions, and most adverse reactions that occur when CMIs and CT are combined are generally counted on CMIs (Peng and Li, 2019). Studies have shown that age ≥60 years, history of allergy, solvent dosage < standard dosage, and drip rate ≥70 drops/min are all independent risk factors affecting the occurrence of adverse drug reaction (ADR) of CMI (Cheng, 2022). In fact, adverse events caused by CMI mostly occur in the elderly population over 60 years of age (Wan and Zhang, 2009; Tan et al., 2014; Zheng et al., 2019), who have a slower metabolism, resulting in a relatively longer retention time of drugs in the body, coupled with reduced liver and kidney function and slower metabolism and excretion of drugs in the elderly, thus more likely to induce adverse drug reactions (Yuan et al., 2021). In order to minimize the occurrence of adverse reactions, clinical application of SMIC requires attention to the following (Peng, 2020): 1) clinical use of drugs follows the principle of “diagnosis and treatment” (Dong and Yao, 2016). 2) Special groups should be administered with caution and their liver and kidney functions should be routinely monitored. 3) The drip rate should not be too fast when administered intravenously. 4) It is advisable to administer the drug alone (Ma, 2015b).

Chinese medicine is rich in philosophical ideas and diagnostic concepts, including holistic, systematic and balanced views, and there is a complementary and reciprocal relationship between these philosophical concepts and biomedical sciences, which can often be used in dealing with the concept of disease in modern medicine (Yang, 2017b; Ma et al., 2017). Angina pectoris arises from the viewpoint of TCM: external factors acting on the human body, manifested as specific changes in emotions, diet, climate, etc., cause an imbalance of yin and yang in the human body, damaging the qi and blood of the heart, making the flow of qi and blood poor, the balance of qi and blood is broken, resulting in chest tightness and pain. Modern medical research has shown (Huang and Luo, 2019) that there are not only a large number of homeostatic regulatory systems in the human body, such as immune enhancement-suppression balance, but also various homeostatic relationships between the human body and the outside world, such as the balance between the normal flora and the immune system, and the balance between body temperature and environmental temperature. These theories of balance, which are similar to the philosophy of TCM, have been gradually confirmed, confirming the connection between the philosophy of TCM and biomedical science.

UA belongs to the category of “thoracic paralysis” in TCM, and its core pathogenesis is paralysis of the heart veins (Zhang and Wu, 2017), while TCM believes that the Danshen has the function of activating blood stasis, removal of blood stasis and generating muscle, which can promote the balance of yin and yang in the body, and has clear clinical efficacy on paralysis of the heart veins (Luo et al., 2021). *The Chinese Pharmacopoeia* 2020 edition records (Chinese Pharmacopoeia Commission, 2020) that *Salvia miltiorrhiza Bge*. contains a variety of pharmacological components, mainly including *Salvia miltiorrhiza* IIA, Salvinone, Salvianolic acid, Salvianolic acid B and Salvianolin, which have the effects of inhibiting platelet aggregation, anti-myocardial ischemia and anti-atherosclerosis. SMIC can alter blood flow velocity and viscosity, improve the local circulatory capacity of patients, improve myocardial function, reduce myocardial cell apoptosis, regulate the expression of apoptosis-related proteins, and modulate the levels of myocardial inflammatory cytokines and oxidative stress products (Luo et al., 2020; Zhao and Liu, 2021). Fufang Danshen injection can inhibit atheromatous plaque formation and maintain the stability of the balance between endothelium-associated mediators such as nitric oxide (NO) and vasoconstrictor endothelin-1 (ET-1) in intimal hyperplasia. It also inhibits lipid peroxidation, improves myocardial tolerance to hypoxia, and improves the metabolic activity of hypoxic myocardium (Chen et al., 2015b). Guanxinning injection has been studied and proven to have antioxidant, inhibit inflammatory response, improve cardiac autonomic regulation, regulate blood lipid and coagulation system, improve cardiac microcirculation (Hu et al., 2017; Guo et al., 2020). Danshenchuanxiongqin injection can increase superoxide dismutase (SOD) and NO levels and reduce malondialdehyde (MDA) levels, thus reducing coronary artery endothelial cell damage, inhibiting thrombus formation, reducing biofilm damage, and playing a certain protective effect on the myocardium (Ding et al., 2018). Danhong injection can increase superoxide dismutase (SOD) activity, alleviate calcium ion overload and reactive oxygen species production (Duan et al., 2015), scavenge lipid peroxidation caused by free radicals, and reduce damage to cardiomyocytes by free radicals (Wang et al., 2010), and also inhibit the overflow of CK and LDH from damaged cardiomyocytes to reduce damage to cardiomyocytes during myocardial ischemia and exert a protective effect on cardiomyocytes (Guo et al., 2008). Danshentong IIA Huangsuanna injection significantly improves collateral circulation and local blood supply in the ischemic area by enhancing coronary blood flow, corrects metabolic disorders in the hypoxic myocardium, improves myocardial resistance to hypoxia, inhibits platelet aggregation and anti-thrombosis (Li et al., 2012; Zhou et al., 2021; Su et al., 2022). Shenxiong Putaotang injection inhibited the increase of TNF-α levels in local and peripheral blood of myocardial tissue caused by ischemia-reperfusion, suppressed the levels of Caspase-12 and ICAM-1 in myocardial cells, and inhibited apoptosis, attenuated the inflammatory response and protected myocardial cells (Li et al., 2014b). Danshenduofensuanyan injection increases serum NO levels, increases SOD activity, decreases MDA levels, and decreases LDH, CK, and CK-MB levels, inhibits damage to myocardium and protects ischemia-reperfused cells (Liu et al., 2010).

Cluster analysis suggested that Danshentong IIA Huangsuanna injection performed better in terms of total effective rate and ECG efficacy, while Danshenchuanxiongqin injection performed better in terms of total effective rate and angina pectoris attacks with weeks as the unit of observation, and Danshenduofensuanyan injection performed better with days as the unit of observation; Shenxiong Putaotang injection performed better in terms of total effective rate and improvement of hs-CRP; Danshentong IIA Huangsuanna injection performed better in terms of improvement of TC and TG.

We noticed that Shenxiong Putaotang has better effect in total effective rate and angina pectoris attacks, total effective rate and hs-CRP improvement and improvement of TC and TG. It showed a better effect of Shenxiong Putaotang on the improvement of each index, and whether this result is related to the fact that Shenxiong Putaotang consists of a variety of native herbs or to the fact that the specific clinical application of Shenxiong Putaotang is more standardized and mature could be an idea for subsequent exploration. The results of the two-by-two comparison suggest that the eight SMIC are better than CT in terms of total effective rate, and the addition of Danshenchuanxiongqin injection and Danhong injection are better than CT treatment in terms of other evaluation indexes.

Our sensitivity analysis showed that the overall results were more robust with 2 weeks as the experimental observation point, and most of the injections were similar to or better than the original results. The addition of Danshenchuanxiongqin injection or Danhong injection was opposite to the original NMA, in addition to the effect of intervention time on the analysis results, it may also be related to the number of included patients, different underlying disease status, etc. It is worth noting that when TC or TG is used as an observation, the results obtained are different from the original results. This led us to note that for lipid-related indicators the results can vary or even appear opposite depending on the time of the intervention; is such a phenomenon only related to the time of onset of SMIC on lipid indicators? Is it possible to follow up this result with a guideline to specify the treatment cycle? It is worth thinking about.

When the trial was limited to a case number ≥90, several results differed from the original meta-analysis, especially regarding blood indicators, including hs-CRP, TC, and TG, and Danshenchuanxiongqin injection and Danhong injection performed differently in these aspects than the original results, possibly because of differences in the number of cases included in each injection and the timing of the intervention. It also suggests that the number of cases is significant for the robustness of the experimental results, and the experimental population should be expanded to obtain results close to the real situation.

This NMA took eight SMIC in clinical practice as a starting point and analyzed and compared their therapeutic effects on patients with unstable angina pectoris. In recent years, the ideology of TCM has become increasingly influential not only in countries around China, but also in countries on other continents. CMI can serve as a good vehicle to inherit and develop the theory and application of TCM. Facing the unstable angina pectoris discussed in this paper, Chinese medicine treatment has certain advantages. Through the summary of the study in this paper, it conveys that SMIC has important value for the treatment of unstable angina, and it is worth to be further promoted to countries where non-Chinese medicine is commonly used, which can become compatible and complementary to modern medicine, and play a role in broadening clinical diagnosis and treatment decisions and providing ideas for new drug development.

The current study provides clues to continue our thinking: in terms of basic research, do different injections do differ in their therapeutic effects, and is it possible to identify the main components that cause the differences? Is the cause of the adverse reaction due to certain ingredients in the injection, the dose used or other reasons? etc. Clinical studies could continue to address these questions: Are there any differences in treatment outcomes between these injections for patients with different severity of disease, and can they be further differentiated for use according to disease? Can it be applied to patients with comorbid other systemic diseases? Can refined evaluation metrics be determined? Etc. All of these issues may become directions for further research at SMIC in the future. The exact therapeutic effect of SMIC deserves more reliable evidence through more in-depth basic research or higher quality clinical studies. We look forward to having more high-quality clinical studies in the future and using NMA to make greater contributions to the research of evidence-based Chinese medicine.

## 5 Limitations

There are some limitations in this study. ① The average quality of the literature included in this paper, the small amount of literature corresponding to individual injections and the insufficient data bring unavoidable data bias. Another part of the reason is that some of the included studies have some methodological shortcomings: no specific methods for random sequence generation and allocation hiding implementations are addressed. ② The RCT studies included in this study were of poor quality in terms of random sequence generation, allocation concealment, and blinding of investigators and subjects, which may have contributed to the presence of selection and measurement bias. ③ The evaluation of adverse reactions in the literature is mostly in terms of symptoms, with fewer reports on safety indicators. ④ The severity of the included patients varied between studies and may have influenced the final outcome metrics. ⑤ Most of the literature has a low number of reports on adverse reactions, which may influence the judgment of adverse reactions.

## 6 Conclusion

In this study, we evaluated eight commonly used SMIC from different outcome metrics and summarized the efficacy of each SMIC in a probabilistic ranking. This NMA showed that Danshenchuanxiongqin injection was the most effective for angina pectoris with a time observation unit of weeks compared to CT. danshentong IIA Huangsuanna injection significantly improved ECG efficacy and, in addition, was the most effective for TG reduction. Shenxiong Putaotang injection was associated with better total effective rate, hs-CRP and TC. Danshenduofensuanyan injection was associated with the efficacy of angina pectoris attacks with days as the time unit of measurement.

In a two-by-two comparison, the addition of Danshenchuanxiongqin injection and Danhong injection outperformed CT treatment alone in all study parameters. Using cluster analysis, the addition of Danshentong IIA Huangsuanna injection performed better when both total effective rate and ECG efficacy were considered; when total effective rate and angina pectoris attacks were considered, Danshenchuanxiongqin injection, Danshenduofensuanyan When considering clinical efficacy and angina pectoris, Danshenchuanxiongqin injection and Danshenduofensuanyan injection were recommended; Shenxiong Putaotang injection performed better in terms of total effective rate and improvement of hs-CRP; regarding the improvement of blood lipids, Danshentong IIA Huangsuanna injection performed better.

Although current studies have confirmed the unique advantages and efficacy of TCM in the treatment of unstable angina, the standardization, safety, and efficacy of clinical applications still need to be studied in depth. In the future, we expect to strengthen the requirements for trial methods; differentiate the degree of patients’ conditions, unify drug doses and more specific safety indicators; and complete higher quality RCT studies so as to further validate the cardioprotective effects of SMIC in UA patients and make efforts for a more standardized and widespread application of SMIC.

## Data Availability

The original contributions presented in the study are included in the article/Supplementary Material, further inquiries can be directed to the corresponding author.

## Abbreviation

NMA, network meta-analysis; ACC, American College of Cardiology; AHA, American Heart Association; CHM, Chinese herbal medicine; TCM, Traditional Chinese Medicine; CMIs, Chinese medicine injections; OCM, Oral Chinese Medicine; SMIC, *Salvia miltiorrhiza* injection classes; RCTs, randomized controlled trials; ACEIs, Angiotensin-converting enzyme inhibitors; ARBs, angiotensin receptor blockers; hs-CRP, high-sensitivity CRP; TC, total cholesterol; TG, triglyceride.
