# Supplementary material for: Efficacy and safety of eight types Salvia miltiorrhiza injections in the treatment of unstable angina pectoris: A network meta-analysis
Source: Front Pharmacol. 2022 Oct 3;13:972738. doi: 10.3389/fphar.2022.972738 (PMC9574204; doi:10.3389/fphar.2022.972738)
Supplement: Supplementary file 1 [file DataSheet1.docx]

**Supplementary materials**

**Supplementary table S1**

The PRISMA checklist of this meta-analysis

| **Section and Topic** | **Item #** | **Checklist item** | **Location where item is reported** |
| --- | --- | --- | --- |
| **TITLE** | | |  |
| Title | 1 | Identify the report as a systematic review. | 1 |
| **ABSTRACT** | | |  |
| Abstract | 2 | See the PRISMA 2020 for Abstracts checklist. | 2 |
| **INTRODUCTION** | | |  |
| Rationale | 3 | Describe the rationale for the review in the context of existing knowledge. | 2-3 |
| Objectives | 4 | Provide an explicit statement of the objective(s) or question(s) the review addresses. | 4-5 |
| METHODS | | |  |
| Eligibility criteria | 5 | Specify the inclusion and exclusion criteria for the review and how studies were grouped for the syntheses. | 6-7 |
| Information sources | 6 | Specify all databases, registers, websites, organisations, reference lists and other sources searched or consulted to identify studies. Specify the date when each source was last searched or consulted. | 7 |
| Search strategy | 7 | Present the full search strategies for all databases, registers and websites, including any filters and limits used. | 8 |
| Selection process | 8 | Specify the methods used to decide whether a study met the inclusion criteria of the review, including how many reviewers screened each record and each report retrieved, whether they worked independently, and if applicable, details of automation tools used in the process. | 8 |
| Data collection process | 9 | Specify the methods used to collect data from reports, including how many reviewers collected data from each report, whether they worked independently, any processes for obtaining or confirming data from study investigators, and if applicable, details of automation tools used in the process. | 9-10 |
| Data items | 10a | List and define all outcomes for which data were sought. Specify whether all results that were compatible with each outcome domain in each study were sought (e.g. for all measures, time points, analyses), and if not, the methods used to decide which results to collect. | 9-10 |
|  | 10b | List and define all other variables for which data were sought (e.g. participant and intervention characteristics, funding sources). Describe any assumptions made about any missing or unclear information. | 9-10 |
| Study risk of bias assessment | 11 | Specify the methods used to assess risk of bias in the included studies, including details of the tool(s) used, how many reviewers assessed each study and whether they worked independently, and if applicable, details of automation tools used in the process. | 9-10 |
| Effect measures | 12 | Specify for each outcome the effect measure(s) (e.g. risk ratio, mean difference) used in the synthesis or presentation of results. | 9-10 |
| Synthesis methods | 13a | Describe the processes used to decide which studies were eligible for each synthesis (e.g. tabulating the study intervention characteristics and comparing against the planned groups for each synthesis (item #5)). | 9-10 |
|  | 13b | Describe any methods required to prepare the data for presentation or synthesis, such as handling of missing summary statistics, or data conversions. | 9-10 |
|  | 13c | Describe any methods used to tabulate or visually display results of individual studies and syntheses. | 9-10 |
|  | 13d | Describe any methods used to synthesize results and provide a rationale for the choice(s). If meta-analysis was performed, describe the model(s), method(s) to identify the presence and extent of statistical heterogeneity, and software package(s) used. | 9-10 |
|  | 13e | Describe any methods used to explore possible causes of heterogeneity among study results (e.g. subgroup analysis, meta-regression). | 9-10 |
|  | 13f | Describe any sensitivity analyses conducted to assess robustness of the synthesized results. | 9-10 |
| Reporting bias assessment | 14 | Describe any methods used to assess risk of bias due to missing results in a synthesis (arising from reporting biases). | 9-10 |
| Certainty assessment | 15 | Describe any methods used to assess certainty (or confidence) in the body of evidence for an outcome. | 9-10 |
| **RESULTS** | | |  |
| Study selection | 16a | Describe the results of the search and selection process, from the number of records identified in the search to the number of studies included in the review, ideally using a flow diagram. | 11 |
|  | 16b | Cite studies that might appear to meet the inclusion criteria, but which were excluded, and explain why they were excluded. | Figure 1 |
| Study characteristics | 17 | Cite each included study and present its characteristics. | Table 1 |
| Risk of bias in studies | 18 | Present assessments of risk of bias for each included study. | 23-24 |
| Results of individual studies | 19 | For all outcomes, present, for each study: (a) summary statistics for each group (where appropriate) and (b) an effect estimate and its precision (e.g. confidence/credible interval), ideally using structured tables or plots. | 12-23 |
| Results of syntheses | 20a | For each synthesis, briefly summarise the characteristics and risk of bias among contributing studies. | 24 |
|  | 20b | Present results of all statistical syntheses conducted. If meta-analysis was done, present for each the summary estimate and its precision (e.g. confidence/credible interval) and measures of statistical heterogeneity. If comparing groups, describe the direction of the effect. | 25-31 |
|  | 20c | Present results of all investigations of possible causes of heterogeneity among study results. | 33 |
|  | 20d | Present results of all sensitivity analyses conducted to assess the robustness of the synthesized results. | 33, Supplementary Figures S3/S4 |
| Reporting biases | 21 | Present assessments of risk of bias due to missing results (arising from reporting biases) for each synthesis assessed. | 34-35 |
| Certainty of evidence | 22 | Present assessments of certainty (or confidence) in the body of evidence for each outcome assessed. | Supplementary figure S2 |
| **DISCUSSION** | | |  |
| Discussion | 23a | Provide a general interpretation of the results in the context of other evidence. | 38-39 |
|  | 23b | Discuss any limitations of the evidence included in the review. | 39 |
|  | 23c | Discuss any limitations of the review processes used. | 40-41 |
|  | 23d | Discuss implications of the results for practice, policy, and future research. | 41-42 |
| **OTHER INFORMATION** | | |  |
| Registration and protocol | 24a | Provide registration information for the review, including register name and registration number, or state that the review was not registered. | Results not obtained |
|  | 24b | Indicate where the review protocol can be accessed, or state that a protocol was not prepared. | 3 |
|  | 24c | Describe and explain any amendments to information provided at registration or in the protocol. | 3 |
| Support | 25 | Describe sources of financial or non-financial support for the review, and the role of the funders or sponsors in the review. | 61 |
| Competing interests | 26 | Declare any competing interests of review authors. | 61 |
| Availability of data, code and other materials | 27 | Report which of the following are publicly available and where they can be found: template data collection forms; data extracted from included studies; data used for all analyses; analytic code; any other materials used in the review. | (61) |

**Supplementary** **table S2** Basic information on the eight kinds of SMICs to be included.

| **Number** | **Generic name** | **Chemical composition** | **Botanical/animal name** |
| --- | --- | --- | --- |
| 1 | Danshen injection | salvia sodium; protocatechuic aldehyde; rosmarinic acid; salvianolic acid B | *Salvia miltiorrhiza* |
| 2 | Fufang Danshen  injection | Phenolic acids; Tanshinone IIA; Cryptotanshinone; Flavonoids; Dihydroflavonoids; Isoflavones | *Salvia miltiorrhiza*; *Dalbergia odorifera* |
| 3 | Guanxinning  injection | Salvianolic acids; Caffeoylquinic acid; Benzene phthalide; Organic acids; Amino acids; Sugar compounds; Protocatechualdehyde; Vanillin | *Salvia miltiorrhiza;* *Ligusticum chuanxiong Hort* |
| 4 | Danshenchuanxiongqin injectio | Salvianol; Caffeic acid; Rosemary acid; Salvianolic acid; Kawarazin | *Salvia miltiorrhiza;* *Ligusticum wallichii* |
| 5 | Danhong injection | Salvianolic acid A; Salvianolic acid B; Tanshinin sodium; Protocatechuic aldehyde; Caffeic acid; Rosemary acid | *Salvia miltiorrhiza;* *Carthamus tinctorius* |
| 6 | Danshentong IIA  Huangsuanna injection | Tanshinone IIA | *Salvia miltiorrhiza* |
| 7 | Shenxiong Putaotang injection | Ligustrazine hydrochloride；Protocatechualdehyd; Caffeic acid ;Danshensu; Lithospermic acid; Salvianolic acid D; Rosmarinic acid; Salvianolic acid A; Salvianolic acid B | *Salvia miltiorrhiza;* *Ligusticum wallichii* |
| 8 | Danshenduofensuanyan injection | salvianolic acid B | *Salvia miltiorrhiza* |

***Supplementary table S3*** The details of SMICs of all the included studies

| Study | Formulation | Source | Species | Quality control reported? (Y/N) | Chemial analysis reported? (Y/N) |
| --- | --- | --- | --- | --- | --- |
| Ren XL 2017 | Danshen injection | Shen Wei  Pharmaceutical Co., Ltd | *Salvia miltiorrhiza* | Y-Drug Specifications Promulgated by the Ministry of Public Health,PR China． Chinese Medicines Formula Preparation Vol 20 | Y-Spectrophotometry |
| Li X 2006 | Danshen injection | Zhengda Qingchunbao Pharmaceutical Co., Ltd | *Salvia miltiorrhiza* | Y-Prepared according to Chinese pharmacopeia | Y-Spectrophotometry |
| Wang XB et al 2013 | Danshen injection | Not mentioned | *Salvia miltiorrhiza* | - | Y-Spectrophotometry |
| Qian YL et al 2000 | Danshen injection | Shanghai No.1 Pharmaceutical Co., Ltd | *Salvia miltiorrhiza* | Y-Prepared according to Chinese pharmacopeia | Y-Spectrophotometry |
| Cheng RQ 2007 | Danshen injection | Not mentioned | *Salvia miltiorrhiza* | Y-Prepared according to Chinese pharmacopeia | Y-Spectrophotometry |
| Zhao QE 2013 | Danshen injection | Not mentioned | *Salvia miltiorrhiza* | - | Y-Spectrophotometry |
| Wang CL et al 2009 | Fufang Danshen injection | Not mentioned | *Salvia miltiorrhiza*; *Dalbergia odorifera* | Y-Prepared according to Chinese pharmacopeia | Y-Spectrophotometry |
| Huang JM 2014 | Fufang Danshen injection | Not mentioned | *Salvia miltiorrhiza*; *Dalbergia odorifera* | Y-Prepared according to Chinese pharmacopeia | Y-Spectrophotometry |
| Zhou H 2010 | Fufang Danshen injection | Not mentioned | *Salvia miltiorrhiza*; *Dalbergia odorifera* | Y-Prepared according to Chinese pharmacopeia | Y-Spectrophotometry |
| WU HL 2019 | Fufang Danshen injection | Chongqing Guotai Canning Pharmaceutical Co., Ltd | *Salvia miltiorrhiza*; *Dalbergia odorifera* | Y-Prepared according to Chinese pharmacopeia | Y-Spectrophotometry |
| Li L 2013 | Fufang Danshen injection | Sichuan Shenghe Lu Medical Pharmaceutical Co., Ltd | *Salvia miltiorrhiza*; *Dalbergia odorifera* | Y-Prepared according to Chinese pharmacopeia | Y-Spectrophotometry |
| Yang T et al 2008 | Guanxinning injection | Not mentioned | *Salvia miltiorrhiza;* *Ligusticum chuanxiong Hort* | - | Y-HPLC |
| Tian ZQ et al 2006 | Guanxinning injection | Shanxi Hengda Pharmaceutical Co., Ltd | *Salvia miltiorrhiza;* *Ligusticum chuanxiong Hort* | Y-Prepared according to Chinese pharmacopeia | Y-HPLC |
| Han YG et al 2013 | Guanxinning injection | Not mentioned | *Salvia miltiorrhiza;* *Ligusticum chuanxiong Hort* | - | Y-HPLC |
| Li XL et al 2012 | Guanxinning injection | Shanxi Yabao Pharmaceutical Co., Ltd | *Salvia miltiorrhiza;* *Ligusticum chuanxiong Hort* | - | Y-HPLC |
| Ma XY et al 2008 | Guanxinning injection | Datong Huida Pharmaceutical Industry | *Salvia miltiorrhiza;* *Ligusticum chuanxiong Hort* | Y-Prepared according to Chinese pharmacopeia | Y-HPLC |
| Tang GX 2015 | Guanxinning injection | Not mentioned | *Salvia miltiorrhiza;* *Ligusticum chuanxiong Hort* | - | Y-HPLC |
| Song ST 2014 | Guanxinning injection | Not mentioned | *Salvia miltiorrhiza;* *Ligusticum chuanxiong Hort* | - | Y-HPLC |
| Meng XH et al 2014 | Danshenchuanxiongqin injection | Guizhou Bite Pharmaceutical Co., Ltd | *Salvia miltiorrhiza;* *Ligusticum wallichii* | Y-Prepared according to Chinese pharmacopeia | Y-HPLC |
| Wang SW et al 2011 | Danshenchuanxiongqin injection | Guizhou Bite Pharmaceutical Co., Ltd | *Salvia miltiorrhiza;* *Ligusticum wallichii* | Y-Prepared according to Chinese pharmacopeia | Y-HPLC |
| Ren YF et al 2012 | Danshenchuanxiongqin injection | Not mentioned | *Salvia miltiorrhiza;* *Ligusticum wallichii* | - |  |
| Yu XL et al 2017 | Danshenchuanxiongqin injection | Jilin Sichang Pharmaceutical Co., Ltd | *Salvia miltiorrhiza;* *Ligusticum wallichii* | Y-Prepared according to Chinese pharmacopeia | Y-HPLC |
| Zhang GY et al 2010 | Danshenchuanxiongqin injection | Guizhou Bite Pharmaceutical Co., Ltd | *Salvia miltiorrhiza;* *Ligusticum wallichii* | Y-Prepared according to Chinese pharmacopeia | Y-HPLC |
| Xu ZJ et al 2022 | Danshenchuanxiongqin injection | Jilin Sichang Pharmaceutical Co., Ltd | *Salvia miltiorrhiza;* *Ligusticum wallichii* | Y-Prepared according to Chinese pharmacopeia | Y-HPLC |
| Cai LL et al 2014 | Danshenchuanxiongqin injection | Guizhou Bite Pharmaceutical Co., Ltd | *Salvia miltiorrhiza;* *Ligusticum wallichii* | Y-Prepared according to Chinese pharmacopeia | Y-HPLC |
| Liu JZ et al 2015 | Danshenchuanxiongqin injection | Not mentioned | *Salvia miltiorrhiza;* *Ligusticum chuanxiong Hort* | - | Y-HPLC |
| Li L et al 2018 | Danshenchuanxiongqin injection | Guizhou Bite Pharmaceutical Co., Ltd | *Salvia miltiorrhiza;* *Ligusticum wallichii* | Y-Prepared according to Chinese pharmacopeia | Y-HPLC |
| Zhang DL et al 2019 | Danshenchuanxiongqin injection | Guizhou Bite Pharmaceutical Co., Ltd | *Salvia miltiorrhiza;* *Ligusticum wallichii* | Y-Prepared according to Chinese pharmacopeia | Y-HPLC |
| Jiang XZ 2019 | Danshenchuanxiongqin injection | Not mentioned | *Salvia miltiorrhiza;* *Ligusticum chuanxiong Hort* | - | Y-HPLC |
| Ren LQ et al 2018 | Danshenchuanxiongqin injection | Guizhou Bite Pharmaceutical Co., Ltd | *Salvia miltiorrhiza;* *Ligusticum wallichii* | Y-Prepared according to Chinese pharmacopeia | Y-HPLC |
| Jiang WB et al 2014 | Danshenchuanxiongqin injection | Guizhou Bite Pharmaceutical Co., Ltd | *Salvia miltiorrhiza;* *Ligusticum wallichii* | Y-Prepared according to Chinese pharmacopeia | Y-HPLC |
| Yang K et al 2014 | Danshenchuanxiongqin injection | Guizhou Bite Pharmaceutical Co., Ltd | *Salvia miltiorrhiza;* *Ligusticum wallichii* | Y-Prepared according to Chinese pharmacopeia | Y-HPLC |
| Li Y 2014 | Danshenchuanxiongqin injection | Not mentioned | *Salvia miltiorrhiza;* *Ligusticum chuanxiong Hort* | - | Y-HPLC |
| Gu GQ 2019 | Danshenchuanxiongqin injection | Guizhou Bite Pharmaceutical Co., Ltd | *Salvia miltiorrhiza;* *Ligusticum wallichii* | Y-Prepared according to Chinese pharmacopeia | Y-HPLC |
| Lan D 2015 | Danshenchuanxiongqin injection | Guizhou Bite Pharmaceutical Co., Ltd | *Salvia miltiorrhiza;* *Ligusticum wallichii* | Y-Prepared according to Chinese pharmacopeia | Y-HPLC |
| Cai LL et al 2013 | Danshenchuanxiongqin injection | Guizhou Bite Pharmaceutical Co., Ltd | *Salvia miltiorrhiza;* *Ligusticum wallichii* | Y-Prepared according to Chinese pharmacopeia | Y-HPLC |
| Li SL 2018 | Danshenchuanxiongqin injection | Guizhou Bite Pharmaceutical Co., Ltd | *Salvia miltiorrhiza;* *Ligusticum wallichii* | Y-Prepared according to Chinese pharmacopeia | Y-HPLC |
| Huang JH 2017 | Danshenchuanxiongqin injection | Jilin Sichang Pharmaceutical Co., Ltd | *Salvia miltiorrhiza;* *Ligusticum wallichii* | - | Y-HPLC |
| Hu XL et al 2014 | Danshenchuanxiongqin injection | Guizhou Bite Pharmaceutical Co., Ltd | *Salvia miltiorrhiza;* *Ligusticum wallichii* | Y-Prepared according to Chinese pharmacopeia | Y-HPLC |
| Zhang YJ 2017 | Danshenchuanxiongqin injection | Shandong Kang Chenxin Pharmaceutical Co., Ltd | *Salvia miltiorrhiza;* *Ligusticum wallichii* | Y-Prepared according to Chinese pharmacopeia | Y-HPLC |
| Yao XD 2015 | Danshenchuanxiongqin injection | Guizhou Bite Pharmaceutical Co., Ltd | *Salvia miltiorrhiza;* *Ligusticum wallichii* | Y-Prepared according to Chinese pharmacopeia | Y-HPLC |
| Li T et al 2018 | Danhong injection | Not mentioned | *Salvia miltiorrhiza;* *Carthamus tinctorius* | Y-Prepared according to Chinese pharmacopeia | Y-HPLC |
| Yu ZB et al 2018 | Danhong injection | Not mentioned | *Salvia miltiorrhiza;* *Carthamus tinctorius* | Y-Prepared according to Chinese pharmacopeia | Y-HPLC |
| Zhou MS et al 2014 | Danhong injection | Not mentioned | *Salvia miltiorrhiza;* *Carthamus tinctorius* | - | Y-HPLC |
| Xu H et al 2011 | Danhong injection | Not mentioned | *Salvia miltiorrhiza;* *Carthamus tinctorius* | - | Y-HPLC |
| Bian ZQ 2015 | Danhong injection | Not mentioned | *Salvia miltiorrhiza;* *Carthamus tinctorius* | - | Y-HPLC |
| Huang Y 2015 | Danhong injection | Not mentioned | *Salvia miltiorrhiza;* *Carthamus tinctorius* | Y-Prepared according to Chinese pharmacopeia | Y-HPLC |
| Liu HX et al 2015 | Danhong injection | Buchang Pharmaceutical Co., Ltd | *Salvia miltiorrhiza;* *Carthamus tinctorius* | Y-Prepared according to Chinese pharmacopeia | Y-HPLC |
| Zhao B 2015 | Danhong injection | Anhui Huayuan Pharmaceutical Co., Ltd | *Salvia miltiorrhiza;* *Carthamus tinctorius* | Y-Prepared according to Chinese pharmacopeia | Y-HPLC |
| Huang HX 2015 | Danhong injection | Not mentioned | *Salvia miltiorrhiza;* *Carthamus tinctorius* | - | Y-HPLC |
| Ma JY 2015 | Danhong injection | Heze Buchang Pharmaceutical Co., Ltd | *Salvia miltiorrhiza;* *Carthamus tinctorius* | Y-Prepared according to Chinese pharmacopeia | Y-HPLC |
| Peng HS et al 2015 | Danhong injection | Not mentioned | *Salvia miltiorrhiza;* *Carthamus tinctorius* | Y-Prepared according to Chinese pharmacopeia | Y-HPLC |
| Yang LJ et al 2016 | Danhong injection | Not mentioned | *Salvia miltiorrhiza;* *Carthamus tinctorius* | - | Y-HPLC |
| Pu YC 2017 | Danhong injection | Heze Buchang Pharmaceutical Co., Ltd | *Salvia miltiorrhiza;* *Carthamus tinctorius* | Y-Prepared according to Chinese pharmacopeia | Y-HPLC |
| Wang Y 2018 | Danhong injection | Not mentioned | *Salvia miltiorrhiza;* *Carthamus tinctorius* | Y-Prepared according to Chinese pharmacopeia | Y-HPLC |
| Pi JB et al 2019 | Danhong injection | Not mentioned | *Salvia miltiorrhiza;* *Carthamus tinctorius* | - | Y-HPLC |
| Lv YY 2016 | Danhong injection | Shandong Danhong Pharmaceutical Co., Ltd | *Salvia miltiorrhiza;* *Carthamus tinctorius* | Y-Prepared according to Chinese pharmacopeia | Y-HPLC |
| Pu DY 2014 | Danhong injection | Jinan Buchang Pharmaceutical Co., Ltd | *Salvia miltiorrhiza;* *Carthamus tinctorius* | Y-Prepared according to Chinese pharmacopeia | Y-HPLC |
| Dai XH 2015 | Danhong injection | Not mentioned | *Salvia miltiorrhiza;* *Carthamus tinctorius* | - | Y-HPLC |
| Chang LD 2021 | Danhong injection | Shandong Danhong Pharmaceutical Co., Ltd | *Salvia miltiorrhiza;* *Carthamus tinctorius* | Y-Prepared according to Chinese pharmacopeia | Y-HPLC |
| Yang XL 2019 | Danhong injection | Shandong Danhong Pharmaceutical Co., Ltd | *Salvia miltiorrhiza;* *Carthamus tinctorius* | Y-Prepared according to Chinese pharmacopeia | Y-HPLC |
| Duan YP 2019 | Danhong injection | Shandong Danhong Pharmaceutical Co., Ltd | *Salvia miltiorrhiza;* *Carthamus tinctorius* | Y-Prepared according to Chinese pharmacopeia | Y-HPLC |
| Liu H et al 2011 | Danhong injection | Not mentioned | *Salvia miltiorrhiza;* *Carthamus tinctorius* | - | Y-HPLC |
| Xia L 2011 | Danhong injection | Not mentioned | *Salvia miltiorrhiza;* *Carthamus tinctorius* | - | Y-HPLC |
| Pu XM et al 2010 | Danhong injection | Not mentioned | *Salvia miltiorrhiza;* *Carthamus tinctorius* | - | Y-HPLC |
| Liu MF 2015 | Danhong injection | Jinan Buchang Pharmaceutical Co., Ltd | *Salvia miltiorrhiza;* *Carthamus tinctorius* | Y-Prepared according to Chinese pharmacopeia | Y-HPLC |
| Yu FL et al 2021 | Danhong injection | Shandong Danhong Pharmaceutical Co., Ltd | *Salvia miltiorrhiza;* *Carthamus tinctorius* | Y-Prepared according to Chinese pharmacopeia | Y-HPLC |
| Jin F et al 2020 | Danhong injection | Shandong Danhong Pharmaceutical Co., Ltd | *Salvia miltiorrhiza;* *Carthamus tinctorius* | Y-Prepared according to Chinese pharmacopeia | Y-HPLC |
| Zhang HL 2015 | Danhong injection | Not mentioned | *Salvia miltiorrhiza;* *Carthamus tinctorius* | - | Y-HPLC |
| Liang K et al 2014 | Danhong injection | Heze Buchang Pharmaceutical Co., Ltd | *Salvia miltiorrhiza;* *Carthamus tinctorius* | - | Y-HPLC |
| Huang MH 2014 | Danhong injection | Shandong Danhong Pharmaceutical Co., Ltd | *Salvia miltiorrhiza;* *Carthamus tinctorius* | Y-Prepared according to Chinese pharmacopeia | Y-HPLC |
| Wang HJ 2012 | Danhong injection | Buchang Pharmaceutical Co., Ltd | *Salvia miltiorrhiza;* *Carthamus tinctorius* | Y-Prepared according to Chinese pharmacopeia | Y-HPLC |
| An YQ 2010 | Danhong injection | Not mentioned | *Salvia miltiorrhiza;* *Carthamus tinctorius* | - | Y-HPLC |
| Pan CK 2012 | Danhong injection | Not mentioned | *Salvia miltiorrhiza;* *Carthamus tinctorius* | - | Y-HPLC |
| Lu BS et al 2015 | Danhong injection | Heze Buchang Pharmaceutical Co., Ltd | *Salvia miltiorrhiza;* *Carthamus tinctorius* | Y-Prepared according to Chinese pharmacopeia | Y-HPLC |
| Yang G 2017 | Danhong injection | Not mentioned | *Salvia miltiorrhiza;* *Carthamus tinctorius* | - | Y-HPLC |
| Liu JL 2016 | Danhong injection | Jinan Buchang Pharmaceutical Co., Ltd | *Salvia miltiorrhiza;* *Carthamus tinctorius* | Y-Prepared according to Chinese pharmacopeia | Y-HPLC |
| Kang HP et al 2015 | Danhong injection | Xianyang Buchang Pharmaceutical Co., Ltd | *Salvia miltiorrhiza;* *Carthamus tinctorius* | Y-Prepared according to Chinese pharmacopeia | Y-HPLC |
| Li K 2018 | Danhong injection | Heze Buchang Pharmaceutical Co., Ltd | *Salvia miltiorrhiza;* *Carthamus tinctorius* | Y-Prepared according to Chinese pharmacopeia | Y-HPLC |
| Li JY 2014 | Danhong injection | Not mentioned | *Salvia miltiorrhiza;* *Carthamus tinctorius* | - | Y-HPLC |
| Li YM et al 2014 | Danhong injection | Not mentioned | *Salvia miltiorrhiza;* *Carthamus tinctorius* | - | Y-HPLC |
| Wang WM et al 2011 | Danhong injection | Not mentioned | *Salvia miltiorrhiza;* *Carthamus tinctorius* | Y-Prepared according to Chinese pharmacopeia | Y-HPLC |
| Xu ZM et al 2016 | Danhong injection | Not mentioned | *Salvia miltiorrhiza;* *Carthamus tinctorius* | - | Y-HPLC |
| Zhang YH 2016 | Danhong injection | Buchang Pharmaceutical Co., Ltd | *Salvia miltiorrhiza;* *Carthamus tinctorius* | Y-Prepared according to Chinese pharmacopeia | Y-HPLC |
| Jiang NX et al 2014 | Danhong injection | Not mentioned | *Salvia miltiorrhiza;* *Carthamus tinctorius* | - | Y-HPLC |
| Xu WD et al 2008 | Danhong injection | Not mentioned | *Salvia miltiorrhiza;* *Carthamus tinctorius* | - | Y-HPLC |
| Xu J et al 2017 | Danhong injection | Heze Buchang Pharmaceutical Co., Ltd | *Salvia miltiorrhiza;* *Carthamus tinctorius* | Y-Prepared according to Chinese pharmacopeia | Y-HPLC |
| Chen SP et al 2019 | Danhong injection | Heze Buchang Pharmaceutical Co., Ltd | *Salvia miltiorrhiza;* *Carthamus tinctorius* | Y-Prepared according to Chinese pharmacopeia | Y-HPLC |
| Song R et al 2021 | Danhong injection | Shandong Danhong Pharmaceutical Co., Ltd | *Salvia miltiorrhiza;* *Carthamus tinctorius* | Y-Prepared according to Chinese pharmacopeia | Y-HPLC |
| Gong FS 2014 | Danhong injection | Heze Buchang Pharmaceutical Co., Ltd | *Salvia miltiorrhiza;* *Carthamus tinctorius* | Y-Prepared according to Chinese pharmacopeia | Y-HPLC |
| Wang XS 2014 | Danhong injection | Not mentioned | *Salvia miltiorrhiza;* *Carthamus tinctorius* | - | Y-HPLC |
| Lai JZ 2012 | Danhong injection | Not mentioned | *Salvia miltiorrhiza;* *Carthamus tinctorius* | - | Y-HPLC |
| Ning WZ et al 2011 | Danhong injection | Not mentioned | *Salvia miltiorrhiza;* *Carthamus tinctorius* | Y-Prepared according to Chinese pharmacopeia | Y-HPLC |
| Gao JB et al 2011 | Danhong injection | Jinan Buchang Pharmaceutical Co., Ltd | *Salvia miltiorrhiza;* *Carthamus tinctorius* | Y-Prepared according to Chinese pharmacopeia | Y-HPLC |
| He WP et al 2012 | Danhong injection | Heze Buchang Pharmaceutical Co., Ltd | *Salvia miltiorrhiza;* *Carthamus tinctorius* | Y-Prepared according to Chinese pharmacopeia | Y-HPLC |
| Lv GY et al 2011 | Danhong injection | Heze Buchang Pharmaceutical Co., Ltd | *Salvia miltiorrhiza;* *Carthamus tinctorius* | Y-Prepared according to Chinese pharmacopeia | Y-HPLC |
| Ju H 2013 | Danshentong IIA Huangsuanna injection | Shanghai No.1 Biochemical Pharmaceutical Co., Ltd | *Salvia miltiorrhiza* | Y-Prepared according to Chinese pharmacopeia | - |
| Li DG et al 2017 | Danshentong IIA Huangsuanna injection | Shanghai No.1 Biochemical Pharmaceutical Co., Ltd | *Salvia miltiorrhiza* | Y-Prepared according to Chinese pharmacopeia | - |
| Huang LY et al 2013 | Danshentong IIA Huangsuanna injection | Not mentioned | *Salvia miltiorrhiza* | Y-Prepared according to Chinese pharmacopeia | - |
| Zhao FL et al 2012 | Danshentong IIA Huangsuanna injection | Shanghai No.1 Biochemical Pharmaceutical Co., Ltd | *Salvia miltiorrhiza* | Y-Prepared according to Chinese pharmacopeia | - |
| Du FD 2018 | Danshentong IIA Huangsuanna injection | Shanghai No.1 Biochemical Pharmaceutical Co., Ltd | *Salvia miltiorrhiza* | Y-Prepared according to Chinese pharmacopeia | - |
| Li XL et al 2016 | Danshentong IIA Huangsuanna injection | Shandong Sishui Aikang Pharmaceutical Co., Ltd | *Salvia miltiorrhiza* | Y-Prepared according to Chinese pharmacopeia | - |
| Han XL et al 2011 | Danshentong IIA Huangsuanna injection | Shanghai No.1 Biochemical Pharmaceutical Co., Ltd | *Salvia miltiorrhiza* | Y-Prepared according to Chinese pharmacopeia | - |
| Chen J 2014 | Danshentong IIA Huangsuanna injection | Shanghai No.1 Biochemical Pharmaceutical Co., Ltd | *Salvia miltiorrhiza* | Y-Prepared according to Chinese pharmacopeia | - |
| Fan ZJ et al 2016 | Danshentong IIA Huangsuanna injection | Not mentioned | *Salvia miltiorrhiza* | Y-Prepared according to Chinese pharmacopeia | - |
| Guo HF 2021 | Shenxiong Putaotang injection | Not mentioned | *Salvia miltiorrhiza;* *Ligusticum wallichii* | Y-Prepared according to Chinese pharmacopeia | Y-HPLC |
| Hu YD 2010 | Shenxiong Putaotang injection | Guizhou Yibai Pharmaceutical Co., Ltd | *Salvia miltiorrhiza;* *Ligusticum wallichii* | Y-Prepared according to Chinese pharmacopeia | Y-HPLC |
| Xi HW et al 2015 | Shenxiong Putaotang injection | Guizhou Jingfeng Pharmaceutical Co., Ltd | *Salvia miltiorrhiza;* *Ligusticum wallichii* | Y-Prepared according to Chinese pharmacopeia | Y-HPLC |
| Li WH et al 2015 | Shenxiong Putaotang injection | Guizhou Yibai Pharmaceutical Co., Ltd | *Salvia miltiorrhiza;* *Ligusticum wallichii* | Y-Prepared according to Chinese pharmacopeia | Y-HPLC |
| He DN 2012 | Shenxiong Putaotang injection | Guizhou Yibai Pharmaceutical Co., Ltd | *Salvia miltiorrhiza;* *Ligusticum wallichii* | - | Y-HPLC |
| Qin QX et al 2010 | Shenxiong Putaotang injection | Guizhou Yibai Pharmaceutical Co., Ltd | *Salvia miltiorrhiza;* *Ligusticum wallichii* | Y-Prepared according to Chinese pharmacopeia | Y-HPLC |
| Chen L 2012 | Shenxiong Putaotang injection | Guizhou Yibai Pharmaceutical Co., Ltd | *Salvia miltiorrhiza;* *Ligusticum wallichii* | Y-Prepared according to Chinese pharmacopeia | Y-HPLC |
| Liang J et al 2011 | Shenxiong Putaotang injection | Guizhou Yibai Pharmaceutical Co., Ltd | *Salvia miltiorrhiza;* *Ligusticum wallichii* | Y-Prepared according to Chinese pharmacopeia | Y-HPLC |
| Luo P et al 2011 | Shenxiong Putaotang injection | Guizhou Yibai Pharmaceutical Co., Ltd | *Salvia miltiorrhiza;* *Ligusticum wallichii* | Y-Prepared according to Chinese pharmacopeia | Y-HPLC |
| Qu Z et al 2011 | Shenxiong Putaotang injection | Guizhou Yibai Pharmaceutical Co., Ltd | *Salvia miltiorrhiza;* *Ligusticum wallichii* | Y-Prepared according to Chinese pharmacopeia | Y-HPLC |
| Liu GD et al 2014 | Danshenduofensuanyan injection | Not mentioned | *Salvia miltiorrhiza* | - | Y-Spectrophotometry |
| Wu YH et al 2010 | Danshenduofensuanyan injection | Shanghai Lvgu Pharmaceutical Co., Ltd | *Salvia miltiorrhiza* | Y-Prepared according to Chinese pharmacopeia | Y-Spectrophotometry |
| Mao YY et al 2016 | Danshenduofensuanyan injection | Shanghai Lvgu Pharmaceutical Co., Ltd | *Salvia miltiorrhiza* | Y-Prepared according to Chinese pharmacopeia | Y-Spectrophotometry |
| Chen F et al 2016 | Danshenduofensuanyan injection | Shanghai Lvgu Pharmaceutical Co., Ltd | *Salvia miltiorrhiza* | Y-Prepared according to Chinese pharmacopeia | Y-Spectrophotometry |
| Chen C et al 2017 | Danshenduofensuanyan injection | Shanghai Lvgu Pharmaceutical Co., Ltd | *Salvia miltiorrhiza* | Y-Prepared according to Chinese pharmacopeia | Y-Spectrophotometry |
| Ren Q et al 2012 | Danshenduofensuanyan injection | Not mentioned | *Salvia miltiorrhiza* | - | Y-Spectrophotometry |
| Han Y et al 2016 | Danshenduofensuanyan injection | Not mentioned | *Salvia miltiorrhiza* | - | Y-Spectrophotometry |
| Liu MY 2017 | Danshenduofensuanyan injection | Not mentioned | *Salvia miltiorrhiza* | - | Y-Spectrophotometry |
| Xia JW et al 2016 | Danshenduofensuanyan injection | Shanghai Lvgu Pharmaceutical Co., Ltd | *Salvia miltiorrhiza* | Y-Prepared according to Chinese pharmacopeia | Y-Spectrophotometry |
| Zhao J 2018 | Danshenduofensuanyan injection | Not mentioned | *Salvia miltiorrhiza* | - | Y-Spectrophotometry |
| Xu J et al 2013 | Danshenduofensuanyan injection | Not mentioned | *Salvia miltiorrhiza* | - | Y-Spectrophotometry |
| Li SL et al 2014 | Danshenduofensuanyan injection | Not mentioned | *Salvia miltiorrhiza* | - | Y-Spectrophotometry |
| Jiao WP et al 2019 | Danshenduofensuanyan injection | Shanghai Lvgu Pharmaceutical Co., Ltd | *Salvia miltiorrhiza* | Y-Prepared according to Chinese pharmacopeia | Y-Spectrophotometry |
| YLDX et al 2016 | Danshenduofensuanyan injection | Not mentioned | *Salvia miltiorrhiza* | - | Y-Spectrophotometry |
| Dou LH 2010 | Danshenduofensuanyan injection | Not mentioned | *Salvia miltiorrhiza* | - | Y-Spectrophotometry |
| Liu YJ 2015 | Danshenduofensuanyan injection | Not mentioned | *Salvia miltiorrhiza* | - | Y-Spectrophotometry |
| Yin YY et al 2013 | Danshenduofensuanyan injection | Not mentioned | *Salvia miltiorrhiza* | - | Y-Spectrophotometry |
| Qin XW 2014 | Danshenduofensuanyan injection | Shanghai Lvgu Pharmaceutical Co., Ltd | *Salvia miltiorrhiza* | Y-Prepared according to Chinese pharmacopeia | Y-Spectrophotometry |
| Xu R et al 2011 | Danshenduofensuanyan injection | Shanghai Lvgu Pharmaceutical Co., Ltd | *Salvia miltiorrhiza* | Y-Prepared according to Chinese pharmacopeia | Y-Spectrophotometry |
| Kang SP et al 2012 | Danshenduofensuanyan injection | Shanghai Lvgu Pharmaceutical Co., Ltd | *Salvia miltiorrhiza* | Y-Prepared according to Chinese pharmacopeia | Y-Spectrophotometry |
| Wang CX 2016 | Danshenduofensuanyan injection | Not mentioned | *Salvia miltiorrhiza* | - | Y-Spectrophotometry |
| Song JJ et al 2017 | Danshenduofensuanyan injection | Not mentioned | *Salvia miltiorrhiza* | - | Y-Spectrophotometry |
| Fan L 2019 | Danshenduofensuanyan injection | Shanghai Lvgu Pharmaceutical Co., Ltd | *Salvia miltiorrhiza* | Y-Prepared according to Chinese pharmacopeia | Y-Spectrophotometry |
| Lei Yan et al 2021 | Danshenduofensuanyan injection | Not mentioned | *Salvia miltiorrhiza* | - | Y-Spectrophotometry |
| Xie YY 2018 | Danshenduofensuanyan injection | Not mentioned | *Salvia miltiorrhiza* | - | Y-Spectrophotometry |
| Qi J et al 2013 | Danshenduofensuanyan injection | Not mentioned | *Salvia miltiorrhiza* | - | Y-Spectrophotometry |
| Yang LL et al 2010 | Danshenduofensuanyan injection | Shanghai Lvgu Pharmaceutical Co., Ltd | *Salvia miltiorrhiza* | Y-Prepared according to Chinese pharmacopeia | Y-Spectrophotometry |
| Xu YH 2016 | Danshenduofensuanyan injection | Not mentioned | *Salvia miltiorrhiza* | - | Y-Spectrophotometry |
| Duan ZX 2016 | Danshenduofensuanyan injection | Shanghai Lvgu Pharmaceutical Co., Ltd | *Salvia miltiorrhiza* | Y-Prepared according to Chinese pharmacopeia | Y-Spectrophotometry |
| Zhang LJ et al 2017 | Danshenduofensuanyan injection | Not mentioned | *Salvia miltiorrhiza* | - | Y-Spectrophotometry |
| Wang Y et al 2016 | Danshenduofensuanyan injection | Not mentioned | *Salvia miltiorrhiza* | - | Y-Spectrophotometry |
| Zhai YY et al 2013 | Danshenduofensuanyan injection | Shanghai Lvgu Pharmaceutical Co., Ltd | *Salvia miltiorrhiza* | Y-Prepared according to Chinese pharmacopeia | Y-Spectrophotometry |
| Wang GL 2017 | Danshenduofensuanyan injection | Shanghai Lvgu Pharmaceutical Co., Ltd | *Salvia miltiorrhiza* | Y-Prepared according to Chinese pharmacopeia | Y-Spectrophotometry |

**
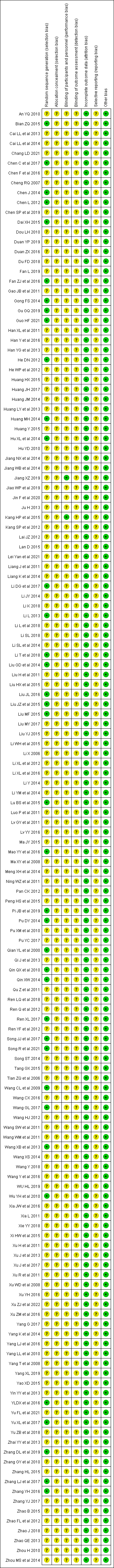
Supplementary figure S1** Summary of results from assessment of studies using the Cochrane risk of bias tool.


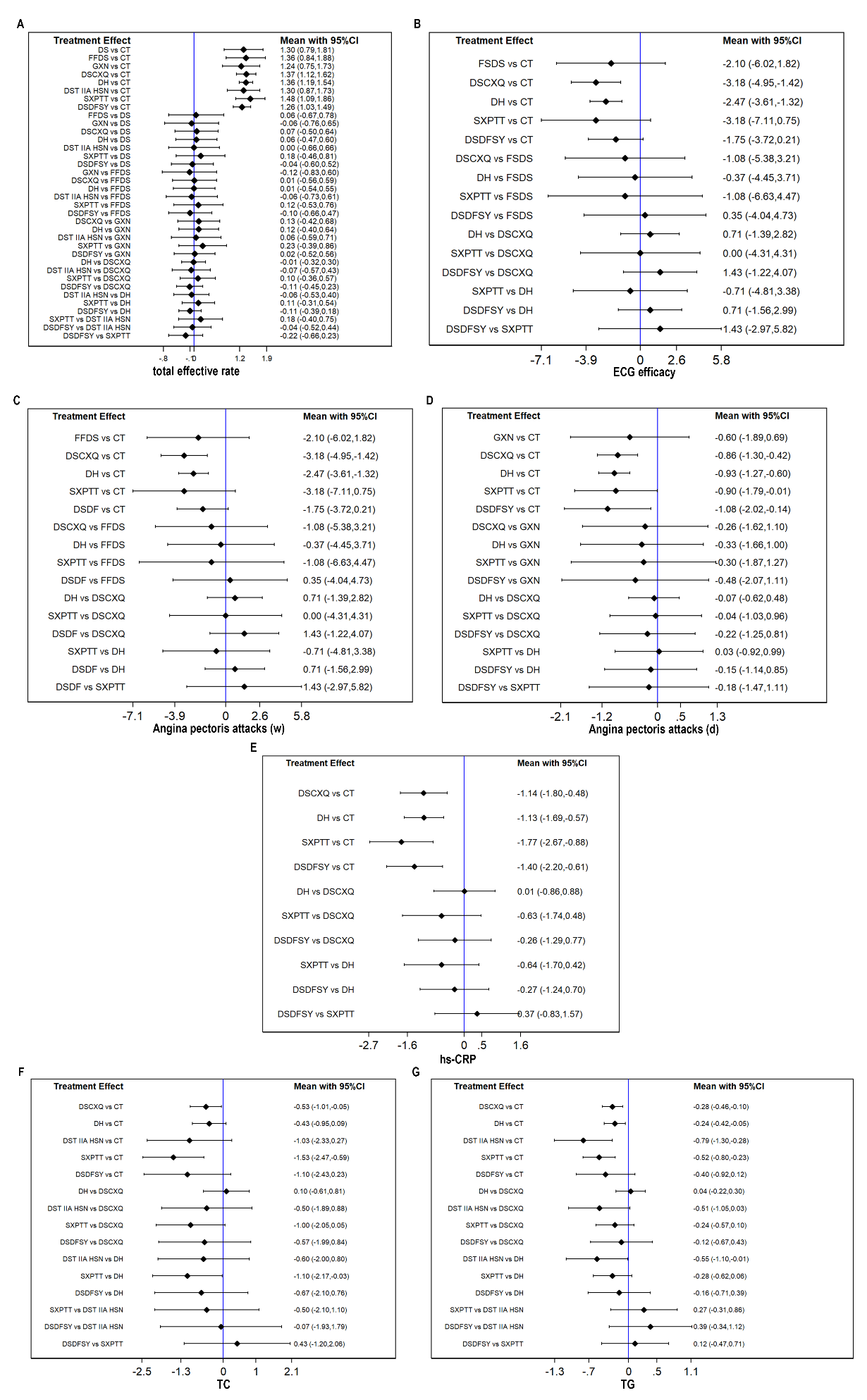


**Supplementary figure S2** Results of a two-by-two comparison of nine treatment modalities

**Supplementary table S3** Pooled estimates of the sensitive analysis (time = 2w).

A.Pooled risk ratios (95% credible intervals) for the total effective rate.

| Danshen injection |  |  |  |  |  |  |  |  |
| --- | --- | --- | --- | --- | --- | --- | --- | --- |
| 1.40  (0.39,4.99) | Fufang Danshen injection |  |  |  |  |  |  |  |
| 0.92  (0.32,2.66) | 0.66  (0.21,2.08) | Guanxinning injection |  |  |  |  |  |  |
| 0.89  (0.36,2.20) | 0.63  (0.23,1.74) | 0.96  (0.47,1.98) | Danshen chuanxiongqin injection |  |  |  |  |  |
| 0.98  (0.41,2.33) | 0.70  (0.26,1.85) | 1.06  (0.54,2.08) | 1.10  (0.74,1.64) | Danhong Injection |  |  |  |  |
| 0.71  (0.27,1.88) | 0.51  (0.17,1.48) | 0.77  (0.35,1.72) | 0.80  (0.45,1.44) | 0.73  (0.43,1.23) | Danshentong IIA Huangsuanna injection |  |  |  |
| 0.87  (0.33,2.27) | 0.62  (0.21,1.79) | 0.94  (0.42,2.08) | 0.98  (0.55,1.74) | 0.89  (0.53,1.49) | 1.22  (0.62,2.40) | Shenxiong Putaotang injection |  |  |
| 1.07  (0.45,2.57) | 0.77  (0.29,2.04) | 1.16  (0.59,2.30) | 1.21  (0.80,1.82) | 1.10  (0.80,1.51) | 1.51  (0.88,2.58) | 1.24  (0.73,2.11) | Danshen duofensuanyan injection |  |
| 3.67  (1.59,8.52) | 2.62  (1.01,6.80) | 3.98  (2.10,7.54) | 4.14  (2.96,5.79) | 3.76  (3.04,4.64) | 5.17  (3.19,8.37) | 4.24  (2.64,6.82) | 3.43  (2.70,4.36) | CT |

B.Pooled risk ratios (95% credible intervals) for the ECG efficacy.

| Guanxinning injection |  |  |  |  |  |
| --- | --- | --- | --- | --- | --- |
| 0.96  (0.38,2.43) | Danshen  chuanxiongqin injection |  |  |  |  |
| 1.04  (0.45,2.39) | 1.08  (0.64,1.83) | Danhong  injection |  |  |  |
| 1.32  (0.51,3.44) | 1.38  (0.68,2.78) | 1.28  (0.72,2.26) | Shenxiong  Putaotang  injection |  |  |
| 0.90  (0.39,2.12) | 0.94  (0.54,1.63) | 0.87  (0.60,1.27) | 0.68  (0.38,1.24) | Danshen  duofensuanyan injection |  |
| 2.70  (1.21,6.01) | 2.81  (1.76,4.50) | 2.60  (2.05,3.31) | 2.04  (1.21,3.44) | 2.99 (2.24,3.99) | CT |

C.Pooled risk ratios (95% credible intervals) for Angina pectoris attacks (w & d). ( week is the bottom left of the assay unit; day is the top right of the assay unit)

| Guanxinning injection | 1.18  (0.28,5.00) | 1.42  (0.35,5.67) | 1.62  (0.31,8.56) | 0.55  (0.14,2.08) |
| --- | --- | --- | --- | --- |
| - | Danshen  chuanxiongqin injection | 1.20  (0.61,2.38) | 1.38  (0.44,4.33) | 0.47  (0.27,0.82) |
| - | 1.74  (0.23,12.91) | Danhong  Injection | 1.14  (0.39,3.33) | 0.39  (0.26,0.57) |
| - | 0.68  (0.09,5.27) | 0.39  (0.08,1.95) | Danshen  Duofensuanyan  injection | 0.34  (0.12,0.92) |
| - | 0.12  (0.02,0.63) | 0.07  (0.02,0.20) | 0.17  (0.05,0.57) | CT |

D. Pooled risk ratios (95% credible intervals) for hs-CRP.

| Danhong  injection |  |  |  |
| --- | --- | --- | --- |
| 2.25  (0.71,7.16) | Shenxiong  Putaotang  injection |  |  |
| 3.90  (1.14,13.39) | 1.73  (0.38,7.78) | Danshen  duofensuanyan  injection |  |
| 0.34  (0.20,0.58) | 0.15  (0.05,0.41) | 0.09  (0.03,0.26) | CT |

E. Pooled risk ratios (95% credible intervals) for TC & TG.( TC is the bottom left of the assay unit; TG is the top right of the assay unit)

| Danshen  chuanxiongqin  injection | 0.96  (0.68,1.35) | 1.71  (0.99,2.95) | 1.32  (0.87,1.99) | 1.16  (0.67,2.01) | 0.78  (0.60,1.00) |
| --- | --- | --- | --- | --- | --- |
| 0.95  (0.57,1.58) | Danhong  injection | 1.79  (1.05,3.05) | 1.38  (0.92,2.05) | 1.21  (0.71,2.08) | 0.81  (0.65,1.02) |
| 1.76  (0.76,4.09) | 1.85  (0.83,4.12) | DanshentongIIA  Huangsuanna  injection | 0.77  (0.43,1.37) | 0.68  (0.34,1.34) | 0.45  (0.28,0.73) |
| 4.20  (1.64,10.76) | 4.41  (1.79,10.87) | 2.39  (0.77,7.36) | Shenxiong  Putaotang  injection | 0.88  (0.49,1.58) | 0.59  (0.43,0.82) |
| 1.89  (0.78,4.59) | 1.98  (0.85,4.63) | 1.07  (0.36,3.17) | 0.45  (0.14,1.44) | Danshen  duofensuanyan  injection | 0.67  (0.41,1.09) |
| 0.63  (0.42,0.94) | 0.66  (0.49,0.89) | 0.36  (0.17,0.75) | 0.15  (0.06,0.35) | 0.33  (0.15,0.74) | CT |

**Supplementary table S4** Pooled estimates of the sensitive analysis (case number ≥90).

A.Pooled risk ratios (95% credible intervals) for the total effective rate.

| Danshen injection |  |  |  |  |  |  |  |  |
| --- | --- | --- | --- | --- | --- | --- | --- | --- |
| 0.88 (0.35,2.24)) | Fufang Danshen injection |  |  |  |  |  |  |  |
| 0.90 (0.31,2.56) | 1.01 (0.37,2.75) | Guanxinning injection |  |  |  |  |  |  |
| 0.80 (0.38,1.70) | 0.90 (0.46,1.78) | 0.89 (0.38,2.05) | Danshen chuanxiongqin injection |  |  |  |  |  |
| 0.85 (0.41,1.76) | 0.96 (0.50,1.84) | 0.95 (0.42,2.14) | 1.07 (0.75,1.53) | Danhong Injection |  |  |  |  |
| 0.75 (0.30,1.89) | 0.85 (0.36,2.01) | 0.84 (0.31,2.25) | 0.94 (0.48,1.84) | 0.88 (0.46,1.67) | Danshentong IIA Huangsuanna injection |  |  |  |
| 0.60 (0.25,1.45) | 0.68 (0.30,1.53) | 0.67 (0.26,1.73) | 0.76 (0.41,1.39) | 0.71 (0.40,1.26) | 0.80 (0.36,1.80) | Shenxiong Putaotang injection |  |  |
| 0.89 (0.42,1.89) | 1.01 (0.51,1.98) | 0.99 (0.43,2.29) | 1.12 (0.74,1.68) | 1.05 (0.74,1.49) | 1.19 (0.61,2.33) | 1.48 (0.81,2.71) | Danshen duofensuanyan injection |  |
| 3.14 (1.57,6.30) | 3.55 (1.92,6.55) | 3.50 (1.60,7.67) | 3.94 (2.94,5.27) | 3.69 (3.00,4.54) | 4.19 (2.28,7.68) | 5.21 (3.06,8.89) | 3.52 (2.65,4.68) | CT |

B.Pooled risk ratios (95% credible intervals) for the ECG efficacy.

| Danshen  injection |  |  |  |  |  |  |  |
| --- | --- | --- | --- | --- | --- | --- | --- |
| 0.96(0.28,3.24) | Fufang  Danshen  injection |  |  |  |  |  |  |
| 1.59(0.40,6.38) | 1.66(0.42,6.47) | Guanxinning injection |  |  |  |  |  |
| 1.17(0.45,3.02) | 1.22(0.49,3.02) | 0.73(0.24,2.27) | Danshen  chuanxiongqin injection |  |  |  |  |
| 1.42(0.57,3.55) | 1.48(0.61,3.56) | 0.89(0.30,2.69) | 1.21 (0.79,1.87) | Danhong  Injection |  |  |  |
| 1.14(0.39,3.34) | 1.19(0.42,3.36) | 0.72(0.21,2.47) | 0.98 (0.48,1.97) | 0.81 (0.42,1.56) | Danshentong IIA Huangsuanna injection |  |  |
| 1.20(0.46,3.12) | 1.25(0.50,3.13) | 0.76(0.24,2.35) | 1.03 (0.62,1.71) | 0.85 (0.54,1.33) | 1.05 (0.52,2.14) | Shenxiong Putaotang injection |  |
| 3.26(1.35,7.86) | 3.40(1.47,7.86) | 2.05(0.70,5.98) | 2.79 (1.97,3.95) | 2.30 (1.78,2.97) | 2.85 (1.55,5.24) | 2.71(1.88,3.92) | CT |

C.Pooled risk ratios (95% credible intervals) for Angina pectoris attacks (w & d). ( week is the bottom left of the assay unit; day is the top right of the assay unit)

| Danshen  chuanxiongqin injection | 1.35 (0.36,5.05) | 0.77 (0.16,3.72) | 0.92 (0.18,4.57) | 0.31(0.12,0.79) |
| --- | --- | --- | --- | --- |
| 0.21(0.01,5.27) | Danhong  Injection | 0.57 (0.12,2.76) | 0.68 (0.14,3.39) | 0.23(0.09,0.59)) |
| - | - | Shenxiong Putaotang injection | 1.20 (0.19,7.44) | 0.41 (0.11,1.46) |
| 0.14(0.00,11.31) | 0.66 (0.02,28.33) | - | Danshen  Duofensuanyan  injection | 0.34 (0.09,1.26) |
| 0.02 (0.00,0.33) | 0.10 (0.02,0.48) | - | 0.14 (0.00,4.30) | CT |

D. Pooled risk ratios (95% credible intervals) for hs-CRP.

| Danshen  chuanxiongqin injection |  |  |  |
| --- | --- | --- | --- |
| 1.06 (0.26,4.34) | Danhong  injection |  |  |
| 1.48 (0.30,7.46) | 1.40 (0.34,5.80) | Danshen  duofensuanyan  injection |  |
| 0.35 (0.11,1.07) | 0.33 (0.14,0.75) | 0.23 (0.07,0.74) | CT |

E. Pooled risk ratios (95% credible intervals) for TC & TG.( TC is the bottom left of the assay unit; TG is the top right of the assay unit)

| Danshen  chuanxiongqin  injection | 1.02 (0.69,1.51) | 1.69 (0.92,3.11) | 0.77 (0.62,0.96) |
| --- | --- | --- | --- |
| 1.20 (0.32,4.54) | Danhong  injection | 1.66 (0.86,3.18) | 0.75 (0.55,1.04) |
| 1.73(0.29,10.26) | 1.44(0.19,10.85) | DanshentongIIA  Huangsuanna  injection | 0.45 (0.26,0.80) |
| 0.62 (0.32,1.20) | 0.52 (0.16,1.64) | 0.36 (0.07,1.87) | CT |

**Supplemantary references:**

[1] Liu, J.，Liu, Y.(2012).Advancement on the pharmacological active constituents of salvia miltiorrhiza.J Liaoning Univ Tradit Chin Med.12( 7) , 15

[2] Xu, Y.，Chen, T.，Chen, M.(2021). Research Progress of Chemical Constituents and Pharmacological Effects of Salvia miltiorrhiza Bunge. Strait Pharmaceutical Journal.33(5) , 45-48.

[3] LH F.， YH W.， GR He.，et al.(2011). Anti-diabetic effect of salvianolic acid A and the possible mechanisms in rats with diabetes mellitus. Chinese Journal of New Drugs.20( 21) , 2063-2068.

[4] He, X., Yang, Y., Zhao, X.S.，et al.(2022). Research Progress on Chemical Constituents and Pharmacological Effects of Dalbergia odorifera [J/OL]. Modern Chinese Medicine.

[5]Ruan, M., Kong, L.Y., Luo, J.G.(2014). Chemical constituents from Guanxinning Injection. Chinese Traditional and Herbal Drugs.45(13) ,1838-1844.

[6]Cao,D.,Huang,X.R.,Liu,Z.T., et al.(2005). Determination of total water-soluble phenolic acids in Salvia miltiorrhiza and Salvia preparations. World Science and Technology.7(4),67-71.

[7]Dong Qinghai. Study on Chemical Constituents and Anti-blood Stasis Effects of Salviae Miltiorrhizae and Ligustrazine Hydrochloride Injection [D]. Jilin University，2020.

[8]Zhou,S.,Chen,T.,Wang,Q.L.(2008). Overview of the pharmacological effects and clinical applications of Danhong injection. Chinese pharmacist.11( 8) , 987-989．

[9]Li,J.R.,Yang,X.,Li,S.Y., et al.(2014). Research progress on the pharmacological effect of Danhong injection. North China Pharmacy.11( 6) , 62-63．

[10]Li,X.,Ju,R.,Li,S.(2021). Research Progress on Chemical Constituents and Pharmacological Effects of Carthamus tinctorius and Predictive Analysis on Quality Markers. Mod Chin Med.23(5) , 928-939.

[11] Gao, X.X.， Sun, Y.， Mi,L.(2018). Effect of Danshen Injection in Improving Symptoms of Myocardial Ischemia. CHINESE ARCHIVES OF TRADITIONAL CHINESE MEDICINE. 36(12) , 3036-3038.

[12] Zhou, L.T.， Tao, L.N.， Qu, X.Y.，et al．(2017).Tissue distribution of two salvianolic acid injections in mice．Chin Hosp Pharm J.37(4),356－359．

[13] Zhang, M.(2020). Clinical Efficacy and Pharmacological Analysis of Rhizoma Ligusticum Glucose Injection in the Treatment of Coronary Heart. Guide of China Medicine.18(9) ,14-15.

[14] Zheng, L. Study on Material Basis for Efficacy and Mechanism of Shenxiong Glucose Injection [D]. Guiyang Medical University,2015.
